# Molecular underpinnings of dedifferentiation and aggressiveness in chromophobe renal cell carcinoma

**DOI:** 10.1172/jci.insight.176743

**Published:** 2024-05-22

**Authors:** Payal Kapur, Hua Zhong, Daniel Le, Ratna Mukhopadhyay, Jeffrey Miyata, Deyssy Carrillo, Dinesh Rakheja, Satwik Rajaram, Steffen Durinck, Zora Modrusan, James Brugarolas

**Affiliations:** 1 Department of Pathology and; 2 Department of Urology, University of Texas Southwestern Medical Center, Dallas, Texas, USA.; 3 Kidney Cancer Program at Simmons Comprehensive Cancer Center, Dallas, Texas, USA.; 4 Lyda Hill Department of Bioinformatics, University of Texas Southwestern Medical Center, Dallas, Texas, USA.; 5 Molecular Biology Department, Genentech Inc., South San Francisco, California, USA.; 6 Hematology-Oncology Division of Internal Medicine, University of Texas Southwestern Medical Center, Dallas, Texas, USA.

**Keywords:** Oncology, Cancer

## Abstract

Sarcomatoid dedifferentiation is common to multiple renal cell carcinoma (RCC) subtypes, including chromophobe RCC (ChRCC), and is associated with increased aggressiveness, resistance to targeted therapies, and heightened sensitivity to immunotherapy. To study ChRCC dedifferentiation, we performed multiregion integrated paired pathological and genomic analyses. Interestingly, ChRCC dedifferentiates not only into sarcomatoid but also into anaplastic and glandular subtypes, which are similarly associated with increased aggressiveness and metastases. Dedifferentiated ChRCC shows loss of epithelial markers, convergent gene expression, and whole genome duplication from a hypodiploid state characteristic of classic ChRCC. We identified an intermediate state with atypia and increased mitosis but preserved epithelial markers. Our data suggest that dedifferentiation is initiated by hemizygous mutation of *TP53*, which can be observed in differentiated areas, as well as mutation of *PTEN*. Notably, these mutations become homozygous with duplication of preexisting monosomes (i.e., chromosomes 17 and 10), which characterizes the transition to dedifferentiated ChRCC. Serving as potential biomarkers, dedifferentiated areas become accentuated by mTORC1 activation (phospho-S6) and p53 stabilization. Notably, dedifferentiated ChRCC share gene enrichment and pathway activation features with other sarcomatoid RCC, suggesting convergent evolutionary trajectories. This study expands our understanding of aggressive ChRCC, provides insight into molecular mechanisms of tumor progression, and informs pathologic classification and diagnostics.

## Abstract 2

Chromophobe renal cell carcinoma dedifferentiates into sarcomatoid, anaplastic, and glandular subtypes, which are associated with increased aggressiveness and metastases.

## Graphical abstract

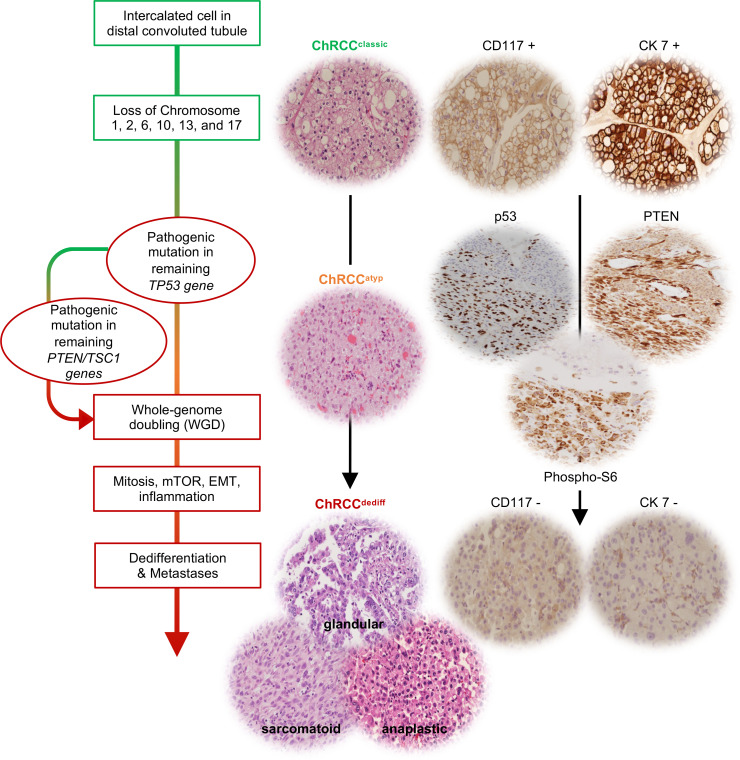

## Introduction

Renal cell carcinoma (RCC) with sarcomatoid mesenchymal change is associated with poor prognosis (1). Sarcomatoid RCC (RCC^sar^) is no longer considered a separate pathological entity and can develop from multiple RCC histologic subtypes (2). However, RCC^sar^ remains a clinically relevant diagnosis due to its increased aggressiveness, difficulty in subtyping when a more differentiated component is absent, resistance to targeted therapies, and increased sensitivity to immune checkpoint inhibitors (ICIs).

The association of poor prognosis with sarcomatoid change is particularly pronounced in chromophobe RCC (ChRCC) (3–5). Though traditionally considered an indolent subtype, ChRCC behaves aggressively when sarcomatoid features are present (3, 4, 6, 7). The World Health Organization /International Society of Urologic Pathology (WHO/ISUP) defines sarcomatoid histology as grade 4 (8). Nevertheless, unlike in other RCC subtypes, histologic grading of ChRCC has not proven prognostic (8).

Based on its microscopic appearance, specifically the presence of cytoplasmic eosinophilia, ChRCC is subdivided into classic (ChRCC^classic^) and eosinophilic (ChRCC^eo^) subtypes. Molecularly, ChRCC, especially the classic variant, is characterized by frequent loss of 1 copy of particular chromosomes (1, 2, 6, 10, 13, and 17) (9, 10). In addition, in one of the first integrated genomic analyses performed, we previously reported that ChRCC is associated with *TP53* and *PTEN* mutations (10). Similar findings were published by The Cancer Genome Atlas (TCGA) (9), which also identified mutations in the *TERT* promoter as well as in mitochondrial DNA.

Recently, 2 manuscripts explored the mutation landscape of metastatic ChRCC. Using a combination of whole genome and targeted region sequencing of metastatic ChRCC cases, Casuscelli et al. found enrichment of *TP53* (58%) and *PTEN* (24%) mutations and duplication of > 3 chromosomes (25%) (11). Roldan-Romero et al. reported mechanistic target of rapamycin (mTOR) pathway gene (*MTOR*, *TSC1*, *TSC2*) mutations and their association with shorter disease-free survival (12).

While both sarcomatoid dedifferentiation and particular mutations have been associated with metastases, how these 2 processes are linked remains unclear. Furthermore, the process whereby a differentiated tumor becomes sarcomatoid remains unknown. To gain insight, we evaluated paired ChRCC samples (epithelial and dedifferentiated) using a comprehensive platform involving whole-exome sequencing (WES) and DNA copy number analyses (CNA), RNA-Seq, and detailed pathological and immunohistochemical studies. This integrated genomic/pathological analysis enabled us to chart a genotype-phenotype evolution of ChRCC. We found that aggressive ChRCC frequently dedifferentiates prior to metastasis along 3 phenotypic paths (sarcomatoid, anaplastic, or glandular dedifferentiation). We identified an intermediate state characterized by the coexistence of epithelial markers with increased mitosis and atypia. Our working model suggests that dedifferentiation is initiated by *TP53* mutation and is followed by whole genome doubling (WGD) of preexisting monosomes and mTOR complex1 (mTORC1) activation.

## Results

### Patient characteristics of aggressive chromophobe RCC.

We interrogated our institutional kidney cancer database for pathologic diagnosis of ChRCC. Between 1998 and 2020, ChRCC was diagnosed in 204 patients from a total of 3,964 consecutive nephrectomies. We identified patients who developed local and/or distant metastasis (henceforth referred to as aggressive ChRCC). At a median duration follow-up of 2.3 years (interquartile range, 0.4–4.8 years), 15 patients developed metastases (7.4%). The patient demographics and baseline tumor characteristics are summarized in Table 1. Demographics (age of nephrectomy, sex, ethnicity, and race) were similar in both cohorts. Tumors that developed metastases were significantly larger (13 cm versus 4.5 cm; *P* < 0.001) and had higher rates of lymphovascular invasion (77% versus 8%; *P* < 0.001) (Table 1).

The clinicopathologic characteristics of 12 aggressive ChRCC with available samples (3 patients underwent resection at outside institutions) are summarized in Supplemental Table 1 (supplemental material available online with this article; https://doi.org/10.1172/jci.insight.176743DS1). Samples from metastasis were available for 9 patients (Supplemental Table 2). None of the patients had documented history of an RCC-associated syndrome, and previously tested patients (*n* = 3) did not have a pathogenic/likely pathogenic germline variant identified. At the time of resection, most aggressive ChRCC were locally advanced. Seven patients had distant metastasis at presentation or within 3 months of diagnosis (M1). Lymph nodes, both regional and distant, were common sites for metastases. At the time of these analyses, 8 patients were deceased due to their disease (information unavailable for 1 patient). Interestingly, all tumors that metastasized had necrosis (100%), and sarcomatoid changes were found in 46% (*P* < 0.001). Overall, these findings suggest that ChRCC metastases develop infrequently but are consistently associated with adverse pathologic features.

### Dedifferentiation occurs frequently in aggressive chromophobe RCC.

**Figure 1:**
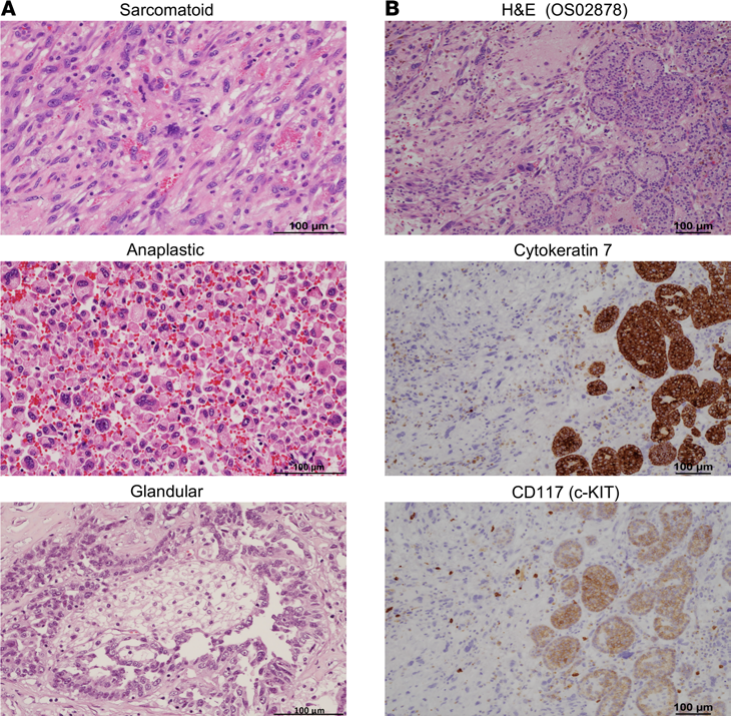
Histologic features of aggressive ChRCC. (**A**) Representative images of H&E-stained slides from aggressive ChRCC tumors showing the 3 dedifferentiation patterns: sarcomatoid, anaplastic, and glandular. (**B**) Representative H&E images of junctional areas between classic and dedifferentiated ChRCC (in OS02878) and corresponding IHC stains showing loss of cytokeratin 7 and CD117 (c-KIT) in ChRCC^dediff^. Scale bar: 100 μm.

We next performed detailed morphological analyses of the 12 aggressive ChRCC with available tissue (all treatment naive samples except for OS03074). Notably, all tumors were of classic subtype. Focal (subclonal) dedifferentiation (ChRCC^dediff^) was observed in 7 (58%) patients. We observed 3 morphologic patterns of dedifferentiation — sarcomatoid, anaplastic, and glandular dedifferentiation (Figure 1A) — and often with more than 1 pattern in the same patient (Supplemental Table 2). The percentage of the sarcomatoid component (spindle cells reminiscent of sarcoma; ref. 2) varied between 5% and 90% and did not show an obvious association with time to recurrence. Anaplastic change comprised sheets of large epithelioid cells with abnormally contoured, pleomorphic, hyperchromatic nuclei; prominent nucleoli; and dense eosinophilic cytoplasm. In 1 case (KC02826), anaplastic dedifferentiation was the only pattern of dedifferentiation (Supplemental Table 2). We report glandular dedifferentiation composed of cuboidal cells in tubules and micropapillae (Figure 1A and Supplemental Figure 1A). Glandular dedifferentiation was the dominant pattern in 2 cases (KC02543 and OS03661), comprising approximately 80% and 60% of the tumor, respectively. CD117 (c-KIT), a marker routinely used for diagnosis of ChRCC shared with the putative cell of origin, was lost in ChRCC^dediff^. Epithelial markers such as cytokeratin 7 (CK7), which are strongly expressed in ChRCC^classic^, were markedly decreased or lost in most ChRCC^dediff^ except for glandular dedifferentiation (Figure 1B, Supplemental Figure 1A, and Supplemental Table 2). Provocatively, in all cases, the transition from classic to dedifferentiation was markedly abrupt.

Tumor samples from 5 patients lacked frank dedifferentiation. On closer evaluation, 3 patients exhibited prominent but focal nuclear atypia (ChRCC^atyp^), which we defined by the presence of prominent nuclear pleomorphism, hyperchromasia, and atypical mitosis. Atypia was particularly striking in the metastatic samples but focally detectable in primary tumors as well (Supplemental Table 2; Group 2 and Supplemental Figure 1A). Taken together, dedifferentiation/atypia was present in all but 2 patients (Supplemental Table 2). Two patients had only classic ChRCC, without readily visible dedifferentiation or atypia in the limited samples available for review, which in 1 case (KC02530) included only regional lymph nodes (Supplemental Table 2; Group 3).

We performed analyses comparing the differentiation state of primary tumors and metastatic samples. Among 9 patients with metastatic samples available for review (Supplemental Table 2), 7 had only dedifferentiated/atypical components in all of their metastatic samples. ChRCC^classic^ histology was observed in only 2 metastatic samples. Given that the dedifferentiation component in primary tumors accounted for approximately 25% (on average), there was substantial overrepresentation of dedifferentiated regions at metastatic sites (*P* = 0.0003). Overall, these data suggest that the dedifferentiated component is more likely to metastasize.

### Transcriptional analyses.

To gain insight into molecular underpinnings of dedifferentiation, we investigated genomic features from paired classic (ChRCC^classic^) and ChRCC^dediff/atyp^ tumor areas (Supplemental Figure 1B and Supplemental Table 2). We first turned to whole transcriptome data from our cohort of 23 aggressive ChRCC (11 patients). Principal component analyses (PCA) identified 3 clusters, ChRCC^classic^, ChRCC^atyp^, and ChRCC^dediff^ (Supplemental Figure 1C). Interestingly, metastatic samples clustered with the corresponding histological subtype rather than separately (Supplemental Figure 1C). These data suggest that the differentiation state has a greater effect on identity than metastasis, suggesting that dedifferentiation, rather than metastasis, is the overarching determinant of biological state.

**Figure 2:**
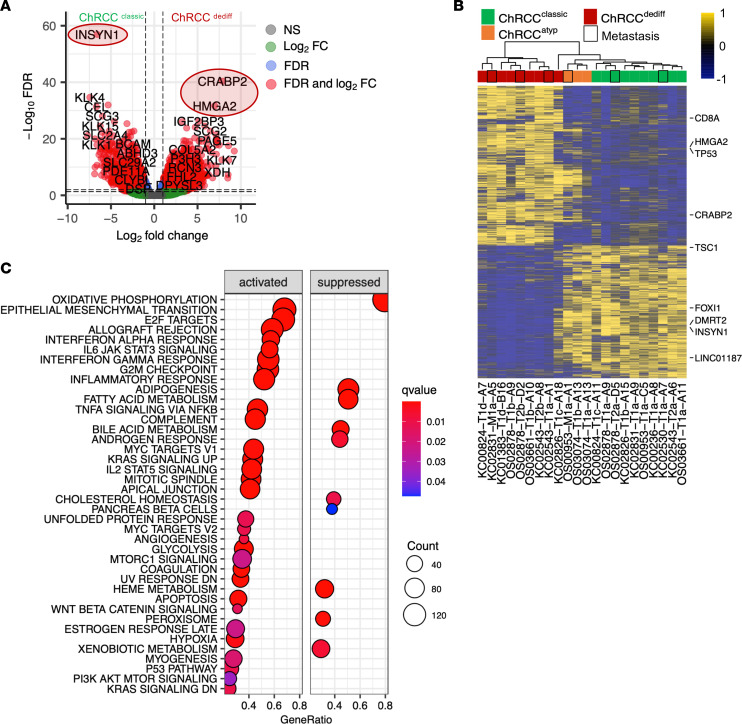
Transcriptomic features of aggressive ChRCC. (**A**) Volcano plot of differentially expressed genes in ChRCC^dediff^ and ChRCC^classic^, color coded according to significance based on fold change and FDR. (**B**) Heatmap of all 4,485 differentially expressed genes (absolute logFC ≥ 1 and FDR ≤ 0.05) between ChRCC^dediff^ and paired ChRCC^classic^ samples from same patients. (**C**) Hallmark gene sets activated and suppressed in ChRCC^dediff^ compared with paired ChRCC^classic^ from same patients.

We explored differential expressed genes (DEG) in ChRCC^dediff^ compared with their paired ChRCC^classic^ samples. We identified a total of 2,441 overexpressed and 2,044 underexpressed genes with an absolute log_2_ fold-change (logFC) ≥ 1 and at a FDR ≤ 0.05 (Figure 2A and Supplemental Table 3). Interestingly, lineage-specific ChRCC signature genes — such as forkhead box I1 (*FOXI1*), its transcriptional target double-sex and mab-3 related transcription factor 2 (*DMRT2*), Rh family C glycoprotein (*RHCG*), and long noncoding RNA *LINC01187* (13) — were all downregulated in ChRCC^dediff^ compared with ChRCC^classic^ (Supplemental Figure 1D).

Differential expression analysis confirmed the findings of the PCA showing that metastatic samples clustered with the corresponding histology and showed that ChRCC^atyp^ clustered between ChRCC^classic^ and ChRCC^dediff^ (Figure 2B). ChRCC^atyp^ shared genes with both ChRCC^classic^ and ChRCC^dediff^, leading us to speculate that it may represent an intermediate stage of tumor progression toward dedifferentiation.

Gene set enrichment analysis (GSEA) demonstrated enrichment in ChRCC^dediff^ for transcriptional pathways involved in epithelial mesenchymal transition (EMT), cell proliferation (E2F targets, G2M checkpoint, mitotic spindle), inflammatory response (IL6/JAK/STAT3, IFN, and TNF signaling), MYC, TP53, and mTOR signaling. On the other hand, catabolic metabolism pathways such as oxidative phosphorylation, fatty acid, and cholesterol homeostasis were downregulated (Figure 2C and Supplemental Table 4). CRABP2 and HMGA2 had the highest expression in ChRCC^dediff^ and have been previously implicated in tumor aggressiveness and EMT (14–16). In contrast, INSYN1 was downregulated and has previously been shown to be associated with indolent RCC (Figure 2A).

To further understand the transcription factor (TF) network, we performed regulon analysis by decoupleR to identify putative TFs with differential activity. Out of 294 TFs, 73 had significantly differential activity between ChRCC^classic^ and ChRCC^dediff^ pairs. As shown in Supplemental Figure 1E, TFs that regulate EMT, stemness, and cell proliferation, such as FOXP2, SNAI1, ZEB2, CREB3, E2F family, TFDP1, and FOXM1, had significantly higher activity in ChRCC^dediff^. Interestingly, a subset of these TF (i.e., E2F1, E2F2, and TFDP1) were already induced in ChRCC^atyp^, suggesting that they may be implicated in the transition from ChRCC^classic^ to ChRCC^atyp^. Conversely, another subset of TFs were exclusively induced in ChRCC^dediff^, suggesting that they may mediate the transition from ChRCC^atyp^ to ChRCC^dediff^ (i.e., FOXP2, SNAI1, ZEB2, CREB3).

### Molecular analyses of paired classic and dedifferentiated samples.

**Figure 3:**
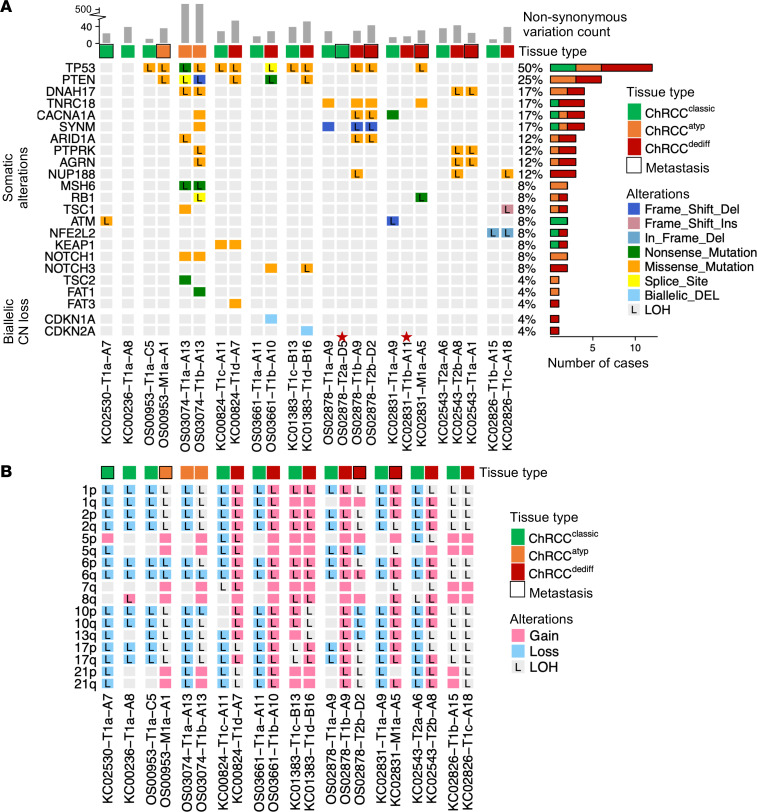
Molecular characteristics of aggressive ChRCC. (**A**) Oncoplot showing frequent somatic (nonsynonymous) alterations in > 2 ChRCC^dediff^ samples (or previously reported in ChRCC) along with variant classification, mutation frequency, and copy number (CN) loss. Each column represents a sample; metastasis are framed in black; L, loss of heterozygosity (LOH). Stars represent samples of low purity and low mutation variant allele frequency (OS02878-T2a-D5, lymph node metastasis; and KC02831-T1b-A11, high intratumoral inflammatory cells). Higher variant number in OS03074 (ChRCC^atyp^) is possibly secondary to *MSH6* mutation. (**B**) Oncoplot of frequent chromosome arm gain or loss. Samples OS02878-T2a-D5, KC02831-T1b-A11, and KC02543-T1a-A1 had low purity and were excluded.

We next turned to WES data from our paired classic (ChRCC^classic^) and ChRCC^dediff/atyp^ tumor areas (Supplemental Table 2 and Supplemental Figure 1B). Classic ChRCC are characterized by nonrandom loss of chromosomes (1, 2, 6, 10, 13, 17 and 21), and we asked how this correlated with dedifferentiation. For these analyses, we evaluated chromosomal copy number changes from our cohort of 24 aggressive ChRCC corresponding to 11 patients (Figure 3B and Supplemental Figure 2). Overall, 8 of 10 evaluable ChRCC^classic^ were hypodiploid, and for the remaining 2 (KC01383 and KC02826), we could not exclude contamination (Supplemental Figure 3A). We observed typical changes in ChRCC^classic^, although loss of chromosome 21 was somewhat less frequent (Supplemental Figure 2). ChRCC^atyp^ exhibited similar findings to ChRCC^classic^ (Figure 3B). Interestingly, when we evaluated the corresponding ChRCC^dediff^ samples, we found that a diploid state had been restored (Figure 3B and Supplemental Figure 2). The simplest explanation for this observation was duplication of the remaining chromosomes, which was observed independently of whether ChRCC^dediff^ samples were from the primary tumor or from metastases, suggesting that chromosomal duplication accompanies dedifferentiation and precedes metastasis development.

In some cases, copy number gains exceeded 2 copies (e.g., chromosome 1q). In fact, all ChRCC^dediff^ showed duplication/gains of multiple chromosomes resulting in aneuploidy (ploidy range, 2–4.35; mean, 2.88) (Supplemental Figure 2C and Supplemental Table 2). Thus, in contrast to the hypodiploid state in ChRCC^classic^, the ChRCC^dediff^ samples underwent amplification of remaining chromosomes through a process that qualifies as WGD, where ≥ 50% autosomal tumor genome showed major allele copy number ≥ 2 (17).

Next, we evaluated total mutation burden (TMB) (nonsynonymous somatic mutations). Median TMB for ChRCC^classic^ was 28 (range, 11–40), which was comparable with previous reports (9) (Supplemental Table 5 and Figure 3A). In contrast, TMB was 40 (range, 26–55) for ChRCC^dediff^. The difference was statistically significant (*t* test, *P* = 0.014).

We asked what may enable WGD and higher mutation rates, and we focused on *TP53*, which is regarded as a guardian of the genome. *TP53* has been previously shown to be mutated in ChRCC by us and others, but how *TP53* mutations correlate with dedifferentiation and metastases is unclear (9–11). We identified somatic mutations in *TP53* in 7 of 11 patients (Figure 3A). *TP53* was mutated in ChRCC^dediff^ in all patients who developed dedifferentiated ChRCC except for 2 (KC02543 and KC02826). Interestingly, *TP53* mutations were also present in ChRCC^atyp^, as well as in a subset of ChRCC^classic^. In every instance, where a mutation was found in ChRCC^classic^, the same mutation was observed in the corresponding ChRCC^dediff/atyp^ sample (Figure 3A, Supplemental Figure 3B, and Supplemental Table 6). Overall, these data suggest that *TP53* mutations arise in ChRCC^classic^ and precede the development of ChRCC^atyp^ and ChRCC^dediff^.

*TP53* mutations are often associated with protein stabilization, which can be evaluated by IHC. Such analyses would also enable us to correlate p53 protein levels with the underlying tumor cell morphology. Interestingly, we observed high nuclear p53 expression in 11 of 12 samples with *TP53* mutations (H-score > 10), suggesting that these mutations had functional consequences (Supplemental Table 2 and Supplemental Figure 3, C and D). Interestingly, in the remaining ChRCC^dediff^, where no *TP53* mutations were found, p53 was similarly stabilized, suggesting that there may be other mechanisms leading to p53 activation. Furthermore, p53 levels often demarcated the transition from ChRCC^classic^ to ChRCC^dediff^ (Supplemental Table 2 and Supplemental Figure 3C). Two samples with chromosome 1p loss and 1q gain (without LOH) did not exhibit mutual exclusivity with *TP53* mutations as described recently (18).

Next, we focused on *PTEN,* which we and others previously identified to be mutated in ChRCC (9, 10). In our series, we observed *PTEN* mutations in 5 patients (Figure 3A and Supplemental Figure 3E). In our cohort, *PTEN* mutations were solely found in ChRCC^dediff/atyp^. PTEN functions as a negative regulator of the PI3K/mTOR pathway, which is also regulated by the TSC1/TSC2 protein complex. Previously, we reported mutations in *TSC1* and *TSC2* in ChRCC (10), and we found mutations in 2 additional samples, an ChRCC^dediff^ and an ChRCC^atyp^, including 1 that did not have a *PTEN* mutation. For 1 patient, we observed 2 different mutations in *TP53* and *PTEN* in 2 ChRCC^atyp^ samples from the same tumor (OS03074), suggesting convergent mutation evolution. Overall, these data are consistent with the notion that *PTEN*/*TSC1*/*TSC2* mutations develop in ChRCC^dediff/atyp^.

**Figure 4:**
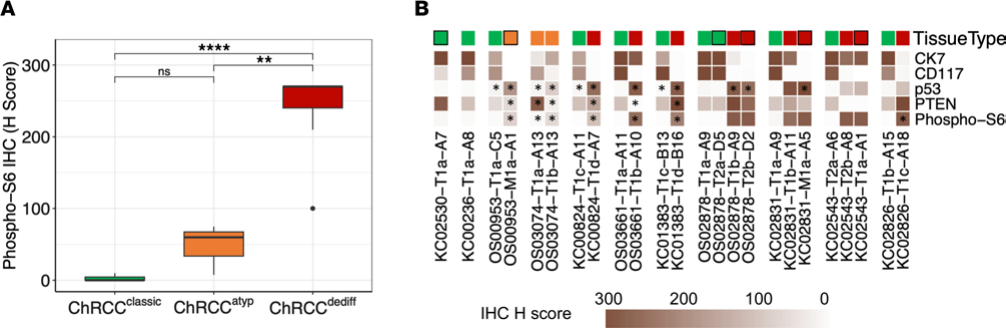
IHC characteristics of aggressive ChRCC. (**A**) Box plot of H-scores (H = intensity [0–3] × percentage of positive cells [0–100]) for cytoplasmic phospho-S6 staining on ChRCC (11 ChRCC^classic^, 3 ChRCC^atyp^, and ChRCC^dediff^). Differential H-score between groups was tested by *t* test. *P* values adjusted using the Benjamini-Hochberg method. **P < 0.01; ****P < 0.0001. (**B**) Heatmap of CK7, CD117, p53, PTEN, and phospho-S6 protein levels in paired ChRCC^classic^ and ChRCC^atyp/dediff^ samples. The asterisks indicate gene mutation present in specimen including *PTEN* or *TSC1*/*2* (Phospho-S6).

**Figure 5:**
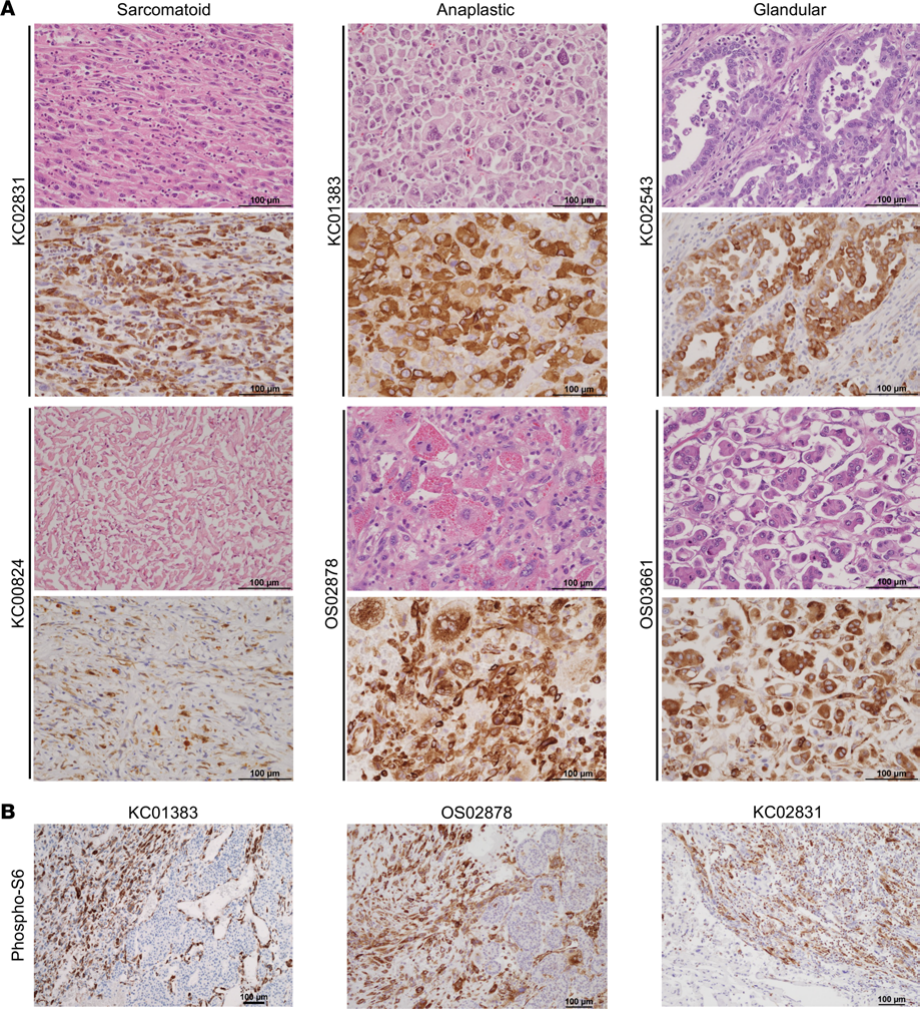
mTORC1 activation in dedifferentiated ChRCC. (**A**) Representative H&E and corresponding phospho-S6 IHC from the 3 patterns of dedifferentiation (sarcomatoid, anaplastic, and glandular), suggesting central role of mTORC1 pathway in ChRCC^dediff^. (**B**) Representative phospho-S6 images illustrating abrupt transition from classic to dedifferentiated ChRCC.

To evaluate the potential effect of *PTEN*/*TSC1*/*TSC2* mutations and integrate the results with the differentiation state of tumor cells, we performed IHC analyses for mTORC1 activation (phospho-S6 Ser240/244). Notably, phospho-S6 was significantly induced in ChRCC^dediff^ (*P* < 0.001; Supplemental Table 2, Figure 4, and Figure 5A). Adjacent areas of ChRCC^classic^ were largely negative (Figure 4A, Figure 5B, and Supplemental Figure 4A), and the transition was typically abrupt and well demarcated (Figure 5B). Increased phospho-S6 was observed in all ChRCC^dediff^, irrespective of the mutation status and type of morphologic dedifferentiation. The 3 samples with atypia had an intermediate degree of phospho-S6 (Supplemental Table 2 and Supplemental Figure 4B).

### Sequence of mutation events.

To further assess the sequence of events, we integrated mutation and CNA. We reasoned that, if mutations preceded the duplication events, they should be found in subsequent chromosomal copies. For these experiments, we overlayed the log odds ratios (logOR) of *TP53* and *PTEN* somatic mutations on the copy number B allele plots (Supplemental Figure 2C).

In 4 patients, we found a *TP53* mutation in the ChRCC^classic/atyp^ component, and all cases had loss of chromosome 17, which resulted in loss of heterozygosity. The same *TP53* mutation was found in the corresponding ChRCC^dediff^, but in ChRCC^dediff^, we found 2 copies of chromosome 17 and the *TP53* mutation was homozygous. Similarly, in the 3 patients where the *TP53* mutation was present only in ChRCC^dediff^, *TP53* mutations were homozygous (Supplemental Figure 2 and Supplemental Table 6). These data suggest that *TP53* mutations developed in ChRCC^classic^ and preceded chromosome 17 duplication.

In contrast to *TP53* mutations, in our series, *PTEN* mutations were only observed in the ChRCC^dediff/atyp^ component. To determine whether they occurred prior to or following chromosome 10 duplication (where *PTEN* lies), we integrated the results with CNA. Interestingly, in every instance, *PTEN* mutations were homozygous, suggesting that they arose before the WGD event.

**Figure 6:**
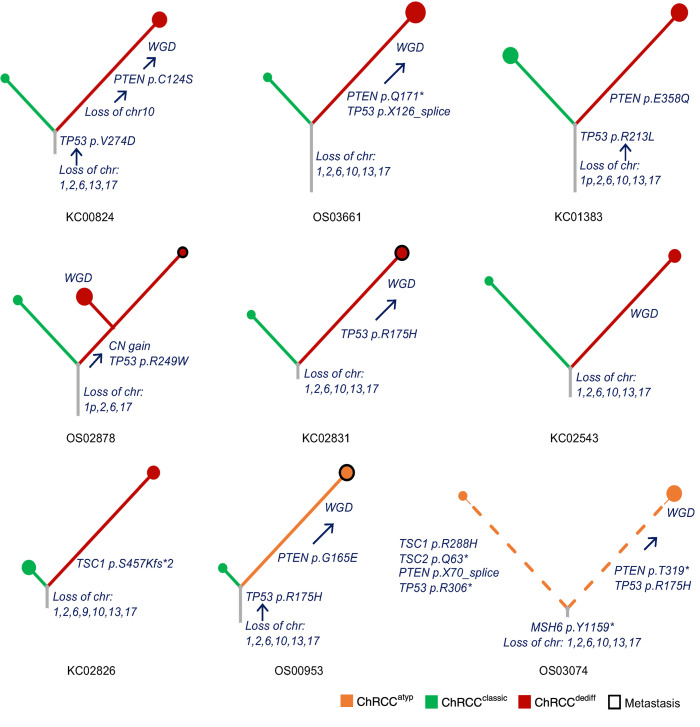
Phylogeny trees. Phylogeny trees colored by ChRCC subcategory of tumor samples from 9 patients based on WES data. Each leaf node represents a sample, metastatic samples are framed in black, and node size indicates ploidy. The length of branch is proportional to the number of unique nonsynonymous somatic mutations (dotted line is used to downscale representation of the high number of variants in OS03074 with *MSH6* mutation). Branches are labeled with putative driver events. WGD, whole genome duplication; CN, copy number.

Finally, we leveraged our unique paired multiregional samples to reconstruct a phylogenic tree for each patient (Figure 6). Allelic copy number loss was detected in the most recent common ancestor (MRCA) for all patients (Supplemental Figure 2 and Supplemental Table 6). Somatic *TP53* mutations were early events, frequently followed by mutations in *PTEN*/*TSC1* and subsequent WGD (Figure 6). Overall, our data suggest that *TP53* mutations, which can be found in ChRCC^classic^ and are hemizygous, precede *PTEN* mutations, which are only found in ChRCC^dediff/atyp^, and are followed by WGD.

### Gene expression reveals converging pathways in dedifferentiated RCC.

**Figure 7:**
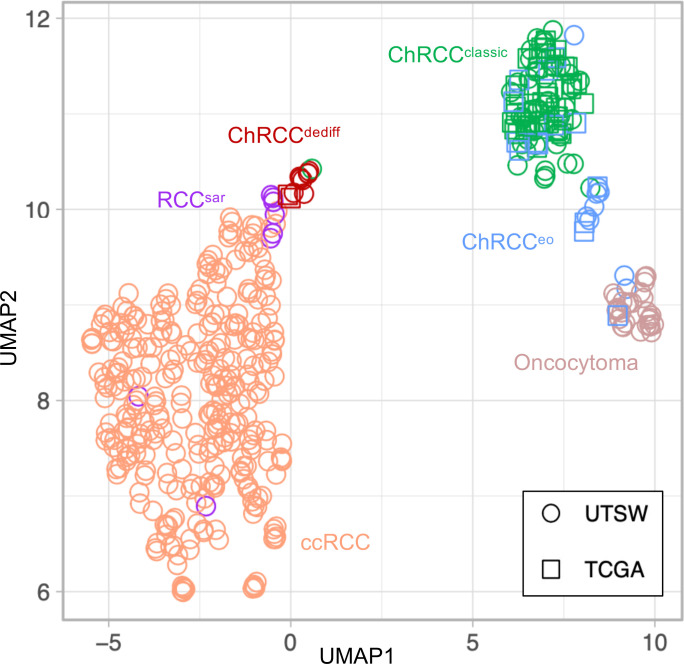
Converging gene expression of ChRCC^dediff^ with other RCC^sar^ subtypes. Uniform Manifold Approximation and Projection (UMAP) of merged RNA-Seq cohorts.

Finally, we thought to extend our gene expression analyses in 2 different ways. First, we sought to expand our ChRCC cohort by including additional samples from TCGA. Second, we sought to determine how sarcomatoid ChRCC (more broadly, ChRCC^dediff^) compares with sarcomatoid differentiation from other RCC histologies. For these experiments, we used previously published data sets from the curated TCGA (KICH) project (9) and UT Southwestern Medical Center (UTSW) (10). We first reviewed the morphology of the KICH cohort using digital slides (https://portal.gdc.cancer.gov), where among 12 ChRCCs reported to have metastasized, we found 2 with a ChRCC^dediff^ component (KN-8427 and KO-8404). We integrated these data with data from UTSW that could be easily harmonized (based on sample processing and data analysis pipeline). Ultimately, our cohort contained ChRCC^dediff^ (9 from UTSW and 2 from TCGA; ref. 9), ChRCC^classic^ (44 from UTSW and 46 from TCGA; refs. 9, 10), ChRCC^eo^ (9 from UTSW and 14 from TCGA; refs. 9, 10), oncocytoma (32 from UTSW; ref. 10), clear cell RCC (ccRCC; 317 from UTSW; refs. 19, 20), and other RCC with sarcomatoid differentiation (RCC^sar^; 8 from UTSW; refs. 19, 20). To minimize batch effect, we utilized normal kidney samples (182 from UTSW and 25 from TCGA; refs. 9, 10) and tumor samples of the same histologic subtype from the different cohorts. Using Uniform Manifold Approximation and Projection (UMAP), we found that the 2 ChRCC^dediff^ from TCGA clustered with the UTSW ChRCC^dediff^. Interestingly, ChRCC^dediff^ tumors clustered away from ChRCC^classic^ and in greater proximity to ccRCC (Figure 7). Furthermore, ChRCC^dediff^ clustered in proximity to 6 of the 8 RCC^sar^, suggesting shared biology (Figure 7). To explore the common variations between RCC^sar^ and ChRCC^dediff^, we focused on the 6 RCC^sar^ that clustered with ChRCC^dediff^. As shown in Supplemental Figure 5, A and B, RCC^sar^ had frequent mutations in *TP53* and mTOR pathway genes. Overall, these findings show that ChRCC^dediff^ share gene expression with other RCC^sar^, suggesting a convergent evolutionary trajectory.

Given the previously noted enrichment of inflammatory response pathways in ChRCC^dediff^, we explored the tumor immune microenvironment in both ChRCC^dediff^ and RCC^sar^. Consistently, we found higher T effector scores in RCC^sar^ and ChRCC^dediff^ relative to ChRCC^classic^ (Supplemental Figure 5C). These findings are compatible with H&E findings of increased inflammatory infiltrates in ChRCC^dediff^ (Figure 5A). This contrasts with ChRCC^classic^, which generally lack an immune infiltrate.

## Discussion

Herein, we sought to probe the process of dedifferentiation and metastasis in ChRCC. ChRCC generally follows an indolent course, and in our series of 204 cases, metastases developed in only 7.4% of the patients. While this frequency is likely an underestimate given our median follow-up of just 2.5 years, other series (5) have shown metastasis development in 5% of patients. Nevertheless, metastatic rates are substantially lower than for ccRCC.

In our series, all tumors that metastasized were of the classic subtype, and there were no ChRCC^eo^. In addition, 60% of the metastatic tumors had dedifferentiation (ChRCC^dediff^). In contrast, dedifferentiation was not observed in any of the nonmetastatic tumors. The frequency of dedifferentiation in our cohort of metastatic ChRCC is higher than reported previously (4). Dedifferentiation may be missed in routine pathological analyses. Furthermore, 2 patients in whom we found only ChRCC^classic^ had limited samples available for review, raising the possibility that dedifferentiation may have been undersampled. Interestingly, while the dedifferentiated clones made up 25% of the primary tumors on average, they represented the exclusive component at sites of metastases in 70% of patients in our cohort. These data suggest that dedifferentiated clones may be more prone to metastasize. In addition, dedifferentiation state, rather than whether the sample was from a primary tumor or a metastasis, was the determinant of overall gene expression, suggesting that dedifferentiation, rather than metastasis, is the overarching determinant of biological state. Thus, while limited by numbers, our data suggest that metastases likely evolve from dedifferentiated aggressive subclones.

We observed 3 morphologic patterns of dedifferentiation: sarcomatoid, anaplastic, and glandular. While both sarcomatoid and anaplastic dedifferentiation have been previously described, to our knowledge, ChRCC glandular differentiation has not been reported. Our data suggest that glandular morphology represents an alternative route of dedifferentiation with a similar endpoint characterized by convergent gene expression and whole genome duplication (WGD). Interestingly, dedifferentiation was typically focal/subclonal, and patterns frequently coexisted in different areas of the same tumor, suggesting some plasticity. Furthermore, the 2 tumors with glandular dedifferentiation also had sarcomatoid change and showed a similar IHC profile except for retained CK7 in the glandular component. Notably, metastases from these 2 patients contained only glandular features, suggesting that glandular dedifferentiation, and not just sarcomatoid change, is associated with aggressiveness.

To probe the underlying biology, we performed multiregion integrated paired pathological and genomic analyses. ChRCC, in particular ChRCC^classic^, is typically hypodiploid with nonrandom loss of chromosomes (chromosomes 1, 2, 6, 10, 13, 17, and 21). In contrast, ChRCC^dediff^ was diploid (or hyperdiploid) with generally 2 or more copies of chromosomes 1, 2, 6, 10, 13, 17, and 21. Though subclonal variation cannot be ruled out, overall, it appears that tumor cells undergo amplification of remaining monosomes to become ChRCC^dediff^ through a process that qualifies as WGD (17), where 50% or more of the tumor genome shows a copy number ≥ 2. Duplication of the remaining chromosomes was observed independently of whether ChRCC^dediff^ samples were from the primary tumor or from metastases, suggesting that chromosomal duplication accompanies dedifferentiation and precedes metastasis development. These findings expand the observations made by Casuscelli et al., who found duplications of ≥ 3 chromosomes (referred to as imbalanced chromosome duplications) in 34.5% (10 of 29) of primary tumors in a cohort of metastatic ChRCC (11).

To understand the transition from ChRCC^classic^ to ChRCC^dediff^, we performed paired mutational analyses. We and others previously showed that *TP53* and *PTEN* are the 2 most commonly mutated genes in ChRCC (9–11). In our cohort of aggressive ChRCC, *TP53* mutations were found in tumors from 64% of the patients. We found *TP53* mutations in both the ChRCC^classic^ and ChRCC^dediff^ components, suggesting that *TP53* mutations are insufficient for ChRCC^dediff^ development. Interestingly, while *TP53* mutations were typically hemizygous in ChRCC^classic^ (*TP53* is on chromosome 17, which is frequently lost), they were homozygous in the corresponding ChRCC^dediff^ component. These data suggest that *TP53* mutations preceded WGD. Similarly, mutations in *PTEN*, which is on chromosome 10, were also homozygous in ChRCC^dediff^. The simplest interpretation for these data is that mutations in *TP53* and *PTEN* precede WGD. Given the role of p53, which has been called “the guardian of the genome” (21), we speculate that *TP53* mutation in ChRCC^classic^ predisposes to WGD and ChRCC^dediff^. ChRCC^dediff^ was also characterized by a higher TMB (40 versus 28 in ChRCC^classic^; *P* = 0.014), which may also be facilitated by *TP53* mutation.

**Figure 8:**
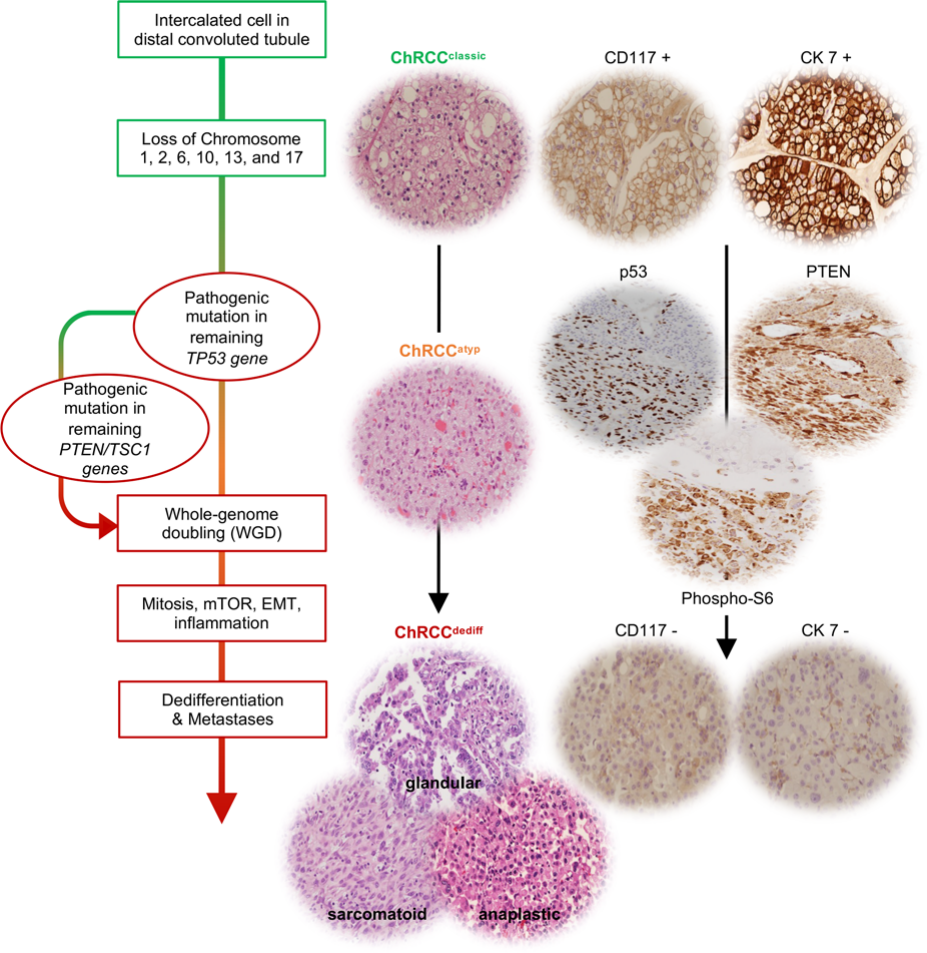
Integrated genomic/pathologic model of evolutionary trajectories. A working model for ChRCC dedifferentiation and metastasis.

Thus, through pathologically guided genomic analyses, we were able to put together a working model for ChRCC dedifferentiation and metastasis (Figure 8). Taken together, our data suggest that metastases often develop through a process of dedifferentiation and EMT, which can present with 3 different morphological patterns and which results from *TP53* and *PTEN* mutation and subsequent WGD. This process is accompanied by mTORC1 activation, which can serve as a biomarker and demarcates dedifferentiated areas.

How mTORC1 becomes activated is not clear. *PTEN* mutations may contribute, but they were only found in a subset of tumors. We and others previously reported the identification of *TSC1*/*TSC2* mutations in chRCC (9–11), but these mutations were also infrequent. While these mutations are likely associated with mTORC1 activation (12), there are probably other mechanisms, as mTORC1 activation (as determined by phospho-S6) was a universal feature of ChRCC^dediff^.

We sought to expand our studies beyond ChRCC and performed gene expression analyses that included non-ChRCC with sarcomatoid differentiation (RCC^sar^). Interestingly, ChRCC^dediff^ tumors clustered away from ChRCC^classic^ and were found in proximity to ccRCC. Furthermore, ChRCC^dediff^ clustered with RCC^sar^. These data suggest that, while sarcomatoid transformation may originate from different RCC histologies, tumors may evolve toward a similar endpoint. In keeping with this notion, there are frequent mutations in *TP53* and *PTEN* in RCC^sar^, which is consistent with previous reports (22).

One feature of sarcomatoid tumors, including ChRCC^dediff^, was inflammation. This was observed by histopathological analyses as well as in GSEA and through analyses of TF underpinning gene expression changes. Pathways included IL-6/JAK/STAT3, IFN-γ, and TNF signaling. One feature of this inflamed TME was higher T effector scores.

This study has practical implications. We found that dedifferentiated ChRCC lacked diagnostic markers such as CD117 and CK7 (except for the glandular component, which retains CK7). This can pose diagnostic challenges. Given the higher rate of ChRCC^dediff^ at metastatic sites compared with primary tumors, our data support prioritizing primary tumors for diagnostic biopsy over metastases, when chRCC is suspected. While this may increase diagnostic accuracy, a potential drawback is an underestimation of dedifferentiated components, which may have implications for therapy. We describe 3 morphological variants of ChRCC^dediff^, sarcomatoid, anaplastic, and glandular. These variants often coexist and are associated with WGD and shared gene expression. They are also characterized by mTORC1 activation. Phospho-S6, a marker of mTORC1 activity, was substantially higher in ChRCC^dediff^ than in other RCCs and demarcates areas of dedifferentiation. In addition, p53 was also commonly induced in ChRCC^dediff^ tumor cells.

Expanding upon prior studies, we found tumor size, advanced pathogenic tumor (pT) stage, presence of sarcomatoid change, tumor necrosis, and lymphovascular invasion to be associated with ChRCC metastasis. Notably, nuclear grade, which is a robust prognostic factor for other RCCs, is not currently recommended for ChRCC (23). This is because nuclear irregularities, prominent nucleoli, and nuclear pleomorphism are ubiquitously present in ChRCC. While new grading systems have been proposed (6, 24, 25), our data support a 3-tier classification. The 3 tiers would involve ChRCC^classic^ (and ChRCC^eo^), ChRCC^atyp^, and ChRCC^dediff^. Our data suggest that ChRCC^atyp^ represents an intermediate step between differentiated and ChRCC^dediff^. Unsupervised gene expression analyses placed ChRCC^atyp^ between differentiated and ChRCC^dediff^. ChRCC^atyp^ shared genes (as well as TFs) with both ChRCC^classic^ and ChRCC^dediff^, suggesting that it represents a transition stage toward dedifferentiation. Similarly, ChRCC^atyp^ exhibited levels of mTORC1 activation that were intermediate between ChRCC^classic^ and ChRCC^dediff^. CNA also placed ChRCC^atyp^ between ChRCC^classic^ and ChRCC^dediff^, with a reduced fraction of chromosome loss and greater gains than ChRCC^classic^ but fewer changes than in ChRCC^dediff^. Morphologically, ChRCC^atyp^ manifested itself by nuclear size variation, hyperchromasia (excluding smudged nuclear atypia that is inherent to oncocytoma and ChRCC), and increased mitosis including atypical mitosis. These findings were often associated with rounding of nuclei, loss of nuclear membrane irregularities (which are inherent to ChRCC^classic^), increased cytoplasmic eosinophilia, and tumor necrosis. Multiinstitutional efforts are ongoing, investigating the value of Ki-67, phospo-S6, and p53 to help recognize the atypical state for routine diagnosis.

Our findings may also have therapeutic implications. In our small series, we observed a complete response to ICI therapy in 1 patient and a partial response in another, both with sarcomatoid change, which suggests that a subset of aggressive ChRCC may be responsive to ICI. Whether ICI responsiveness is similar among anaplastic and glandular subtypes remains to be determined. The similar inflammatory infiltrate and convergent gene expression suggest that these other dedifferentiated subtypes may also be responsive to ICI. However, further research is required and metastatic ChRCC appear to be particularly resistant to ICI compared with other histological subtypes. In addition, one of the patients in our series, a patient with a *TSC1* truncating mutation, had substantial benefit from everolimus, an mTORC1 inhibitor.

This study has several limitations. Foremost is the limited number of patients studied, which is due to the low frequency of ChRCC and, in particular, ChRCC^dediff^. In addition, we were also limited in the number of RCC^sar^ that could be included due to challenges associated with harmonization of different data sets. Nevertheless, our results support a working model that can be tested in other cohorts.

In summary, through comprehensive genomic and transcriptomic studies including comparative studies from morphology-driven multiregional sampling, we provide insight into molecular mechanisms underlying dedifferentiation and metastasis in ChRCC with clinical implications.

## Methods

### Sex as a biological variant.

Sex was not considered as a biological variable.

### Case selection and clinical data extraction.

We searched our institutional RCC database of 3,964 consecutive partial and/or radical nephrectomies from 3,728 patients (between 1998 and 2020 at UTSW and between 2003 and 2017 at Parkland Hospital, Dallas, Texas, USA) to identify nephrectomies with pathologic diagnosis of ChRCC as described previously (26, 27). Relevant clinical data were collected from the database and complemented through comprehensive review of electronic medical records. The tumors were staged based on the 2018 American Joint Committee on Cancer (AJCC) TNM classification for pathologic staging.

### Morphologic evaluation.

Where available, archival material was retrieved and rereviewed by a genitourinary (GU) pathologist to confirm the histology. For each patient, all available metastases samples were thoroughly evaluated for sarcomatoid change and dedifferentiation. Dedifferentiation was defined per previous recommendations (28) as the presence of any component of markedly atypical neoplastic cells with hyperchromasia and pleomorphism, including spindle cells (sarcomatoid features), anaplastic monomorphic round cells, and pleomorphic giant cells (anaplastic features). For a small subset of metastatic RCC cases, outside institution nephrectomy slides were reviewed at the time of presentation to our institution; however, slides and blocks were not available for further analyses.

### IHC.

Supportive IHC analyses were performed, if needed, at our kidney cancer program core histology lab on representative 3–5 μm formalin-fixed paraffin-embedded (FFPE) whole tumor tissue sections. IHCs included CK7 (M7018-OV-TL 12/30, 1:100; Dako), CD117 (A4502, 1:700; Dako), PTEN (7196-A103, 1;200; Dako), p53 (37259-3F9, 1:100; Abcam), vimentin (M0725, 1:75; Dako), and phospho-S6 (Ser240/244) (5364-D68F8, 1:300; Cell Signaling Technology). IHC was performed using a Dako automated system (Agilent). Phospho-S6, p53, and PTEN expression was determined based on the percentage of tumor cells staining positive (0%–100%) and the intensity of expression (range, 0–3), which were multiplied (H-score).

### Nomenclature and annotation.

A unique patient ID was assigned to each patient in our kidney cancer database. Samples used for genomic sequencing are labeled with a “Sample ID” using a nomenclature format that can identify samples from the same patient and the source of the tumor (N, normal; T, tumor; M, metastasis). Samples are numbered in the sequential order they are collected (thus, T1a in the nomenclature does not refer to stage of the tumor).

### Next generation sequencing.

All H&E stained FFPE slides were examined to select the most representative areas from paired classic (ChRCC^classic^) and dedifferentiated (ChRCC^dediff^) areas from primary and metastatic tumors (when available), and matched benign kidney was included. To enable integrative genomic analyses, the corresponding areas were punched (i.e., classic and dedifferentiated areas from primary tumors and metastases as well as from benign kidney), and both DNA and RNA were simultaneously extracted from the same specimen as described previously (29). DNA and RNA-Seq werperformed as previously described (10). DNA-Seq was performed using 75 bp paired-end fragments at an average read depth > 100× on a HiSeq2500 platform (Illumina). On average, 50 million reads per sample were obtained, for RNA-Seq using 50 bp single-end on a HiSeq2500 platform.

### Somatic mutation calling from WES.

WES reads from FASTQ files were aligned to the human reference genome GRCh38 (hg38) using BWA algorithm (version 0.7.15-r1140) set to default parameters (30). Picard (version 2.18) was used to mark PCR duplicates. GATK toolkit (version 4.1.4.1) (31–33) was used to perform base quality score recalibration and local realignment around indels. Strelka2 (version 1.0.15) (34) was used to call somatic variants and small-scale insertions and deletions (indels) for each pair of tumor and normal samples. ANNOVAR was used to annotate somatic mutations and indels (35). Ensembl Variant Effect Predictor (VEP, release 99) (36) was used to assign putative functional consequences. Variants were classified according to the American College of Medical Genetics and Genomics (ACMG) 2015 Guidelines. cBioPortal (https://www.cbioportal.org/), COSMIC (https://cancer.sanger.ac.uk/cosmic) and ClinVar (www.ncbi.nlm.nih.gov/clinvar/) were used to annotate cancer relevance and clinical potential for detected variants. A variant allele frequency (VAF) ≥ 15% in tumor samples was required to call a somatic variant. Intronic, splice regions, untranslated regions, and intergenic and silent mutations were filtered out. The somatic mutation hotspots were identified based on a published resource for statistically significant mutations in cancer (version V2) (37). Two samples with low purity estimate and low overall VAF were excluded for mutation analyses (OS02878-T2a-D5 and KC02831-T1b-A11). Somatic variants reported in the oncoplot were, in addition, individually inspected using Integrated Genomics Viewer (IGV; version 2.13.1; Broad Institute, MIT Harvard, Cambridge, Massachusetts, USA) (38).

### Somatic copy number calling from WES.

Somatic allelic copy number variation (CNV) analyses were carried out on paired tumor and normal WES samples using FACETS (version 0.6.2) (39) and FACETS-suite (version 2.0.8) R packages. Based on human common variation sites from the Single Nucleotide Polymorphism Database (dbSNP) (40), FACETS evaluated read coverage of chromosomal segments and estimated purity, ploidy, and total and allelic integer copy number for tumor samples. The FACETS-suite provided a wrapping function to the FACETS algorithm enabling a 2-pass run to calculate overall copy number and sample purity first and to then detect more focal events with increased sensitivity. The 2 passes were performed with critical values set to 1,000 and 500, respectively, to tune the coarseness of chromosome segmentation. Plots of copy number log ratio, B allele log odds ratios (logOR), and integer copy number were produced by the FACETS-suite. We manually calculated the logOR of *TP53* and *PTEN* somatic mutations, under the assumption that the mutations were on the major allele, and we overlayed them on the B allele plots. By matching gene locations, gene-level integer copy numbers were inherited from the segment integer copy number from the second pass run. Chromosome arm level gain or loss was called when > 50% of the chromosome arm had copy number gain or loss. Differential gene-level copy number comparisons between paired ChRCC^dediff^ and ChRCC^classic^ to identify significantly amplified genes used a *t* test adjusted *P* ≤ 0.05 and integer copy number FC ≥ 2. These analyses excluded 3 samples with purity estimate < 0.3 or significant artifacts (OS02878-T2a-D5, KC02831-T1b-A11, and KC02543-T1a-A1).

### Gene expression analyses.

RNA-Seq raw data were analyzed using HTSeqGenie (41) from Bioconductor. Reads with low nucleotide qualities (70% of bases with quality ≤ 23) or matched to rRNA were removed prior to alignment. Adapter sequences were similarly removed. The remaining reads were aligned to the human reference genome (version GRCh38.p10) using GSNAP (42, 43) (version 2013-10-10-v2), with parameters: “-M 2 -n 10 -B 2 -i 1 -N 1 -w 200000 -E 1 --pairmax-rna=200000 --clip-overlap” and maximum of 2 mismatches per 75 base sequence. Transcripts were annotated based on the Gencode (44) human genes database (version 27). Gene expression levels were quantified by the number of reads mapped unambiguously to the exons of each gene using FeatureCounts (45). Gene counts in tumor samples were filtered using the R package edgeR (version 3.38.4) (46) by requiring at least 10 read counts in at least 2 tumor samples. Normalization was conducted among all high-quality tumor RNA-Seq samples in this cohort using the trimmed mean of M values (TMM) (47) algorithm from edgeR. PCA plots were generated using all filtered genes among tumor samples. Differential gene expression analysis was performed by edgeR to identify DEGs (absolute logFC ≥ 1 and FDR ≤ 0.05) between ChRCC^dediff^ and ChRCC^classic^ on a subcohort from 7 patients with paired samples. GSEA was deployed by clusterProfiler (48) (version 4.4.4) on basis of the logFC values (ChRCC^dediff^/ChRCC^classic^) from DE analysis to identify enriched MSigDB Hallmark gene sets (49, 50) (*q* ≤ 0.05). Regulon analysis was performed using Bioconductor package decoupleR (51) to extract the activity of TF from expression data. Differential TF activity was tested on quantified activity levels by *t* test between ChRCC^dediff^ and ChRCC^classic^ for each TF. The T effector score for each tumor sample was calculated by the mean logCPM (transformed from normalized gene read counts by voom; ref. 52) of the T effector signature genes reported (53). In addition, we integrated the current cohort (9 ChRCC^dediff^, 10 ChRCC^classic^, and 9 normal) with a prior UTSW cohort (32 oncocytoma, 34 ChRCC^classic^, 9 ChRCC^eo^, 317 ccRCC, 8 RCC^sar^, and 173 normal) (10, 19, 20) and the TCGA-curated KICH cohort (46 ChRCC^classic^, 14 ChRCC^eo^, 2 ChRCC^dediff^, and 25 normal) (9). Bioconductor packages edgeR and sva (version 3.44.0) (54) were used to perform normalization and batch effect minimization (using ComBat; ref. 55) on count data. UMAP (56) plots were generated by R package umapr (version 0.0.0.9001) (57) using all filtered genes with default parameters. The T effector scores for UTSW tumor samples were calculated by the mean logCPM expression of the T effector signature genes reported (53). The logCPM expression was transformed from normalized gene read counts by voom (52) on the merged data set.

### Driver phylogenic tree reconstruction.

For each of the 9 patients with ≥ 2 tumor samples, we used nonsynonymous somatic mutations with VAF ≥ 15% to manually reconstruct phylogenic trees to infer the progression. Each leaf node represents a sample that is colored by ChRCC subcategory, and the corresponding node size indicates ploidy. Nodes corresponding to metastases were framed in black. Branches were colored by the ChRCC subcategory of the child nodes to represent common variations. The length of the branch is proportional to the number of common/unique nonsynonymous somatic mutations. Each branch was labeled with putative driver events, including both mutations and copy number changes.

### Statistics.

All statistical analyses were conducted using R (v4.2). Unless otherwise stated, all comparisons for continuous variables were performed using a 2-tailed *t* test (R function t.test) for 2 groups. *P* < 0.05 was considered significant. For all box plots, the horizontal line represents the median; the lower and upper hinges correspond to the first and third quartiles, respectively.

### Study approval.

The study was conducted with approval by the UTSW IRB (STU 02215-015).

### Data availability.

Values for all data points in graphs are available in the Supporting Data Values file. Sequencing files for patients with explicit consent to share genomic information are available in the European Genome-Phenome Archive (study ID: EGAS50000000287; WES dataset: EGAD50000000415; RNA-Seq: EGAD50000000416).

## Author contributions

Conceptualization was contributed by PK and JB; histology and phenotype characterization were contributed by PK; sample acquisition and nucleic acid extraction were contributed by RM, DC, JM, and PK; sequencing was contributed by DL and ZM; genomic/transcriptomic analysis was contributed by HZ, DL, SD, ZM, PK, and JB; IHC was contributed by JM, DC, and PK; data curation was contributed by HZ, DL, SD, ZM, PK, and JB; writing of the original draft was contributed by PK; review and editing were contributed by JB, HZ, and PK; resources were contributed by DR, ZM, SR, PK, and JB; supervision was contributed by PK, ZM, and JB; and funding acquisition was contributed by PK.

## Supplementary Material

Supplemental data

Supplemental tables 1-4

Supporting data values

## Figures and Tables

**Table 1:**
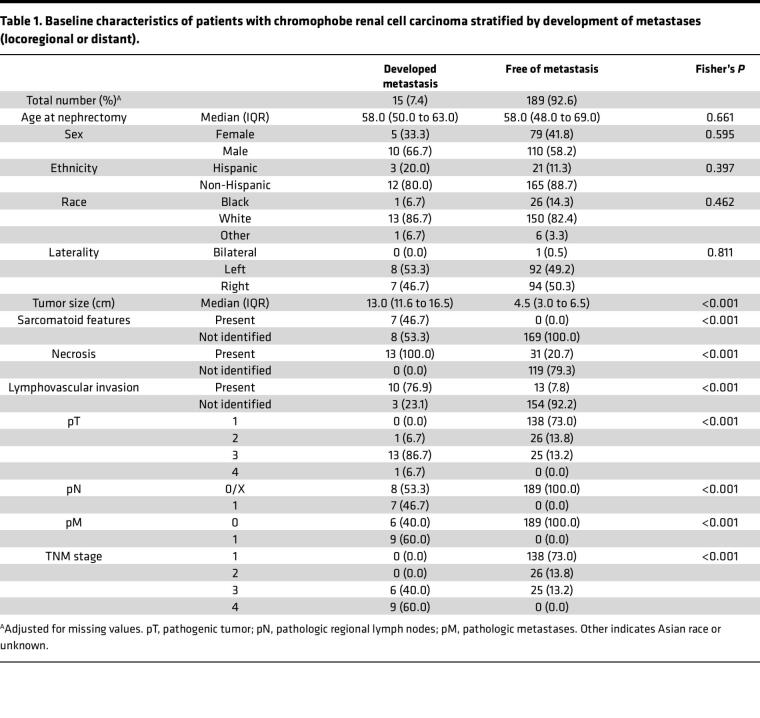
Baseline characteristics of patients with chromophobe renal cell carcinoma stratified by development of metastases (locoregional or distant).

## Version 1 05/22/2024

Electronic publication
