# Supplementary material for: Molecular underpinnings of dedifferentiation and aggressiveness in chromophobe renal cell carcinoma
Source: JCI Insight. 2024 May 22;9(10):e176743. doi: 10.1172/jci.insight.176743 (PMC11141915; doi:10.1172/jci.insight.176743)

Figure S1

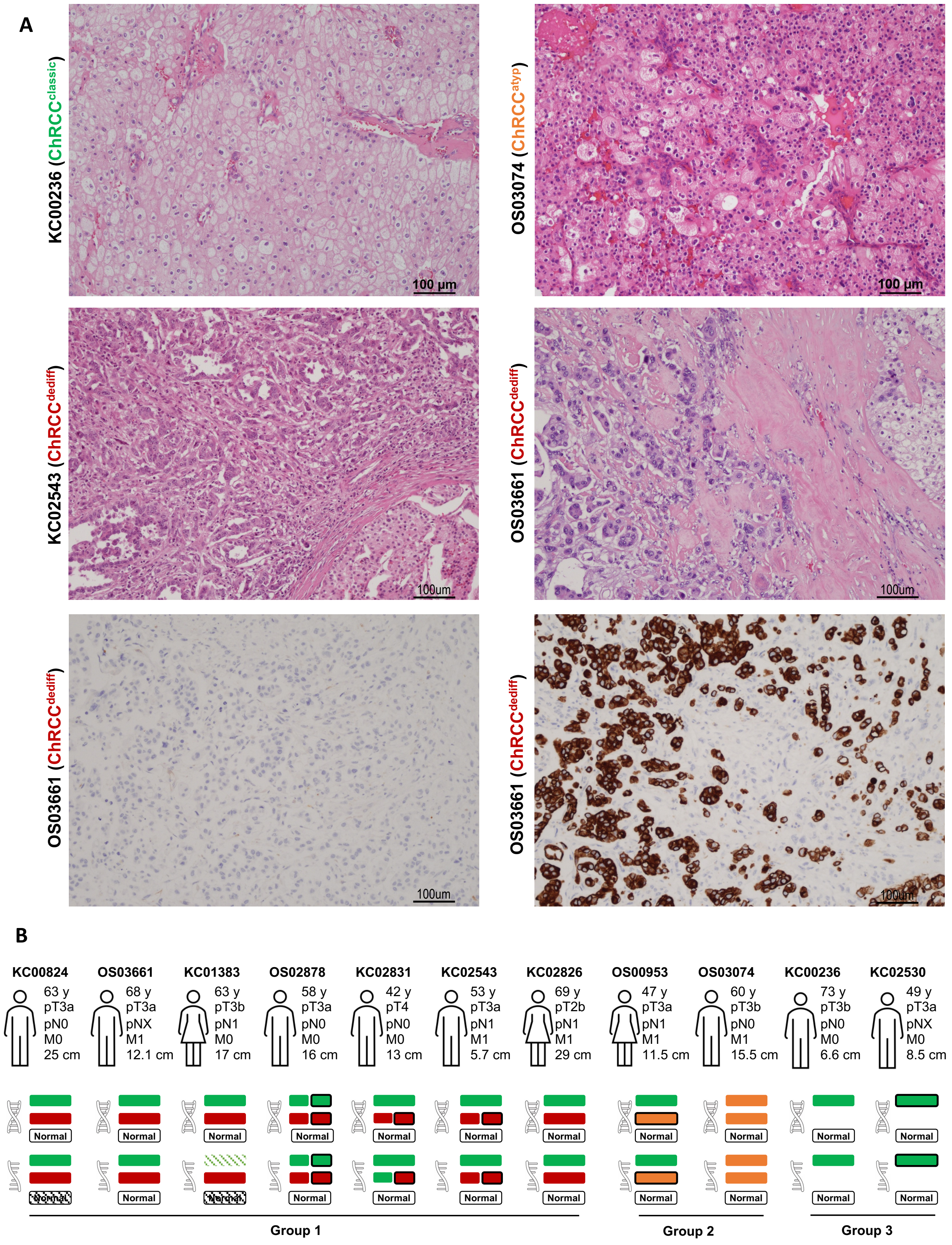
A
KC00236 (ChRCC^classic)
OS03074 (ChRCC^atyp)
100 µm
100 µm
KC02543 (ChRCC^dediff)
OS03661 (ChRCC^dediff)
100um
100um
OS03661 (ChRCC^dediff)
OS03661 (ChRCC^dediff)
100um
100um
B
KC00824
63 y pT3a pN0 M0 25 cm
OS03661
68 y pT3a pNX M1 12.1 cm
KC01383
63 y pT3b pN1 M0 17 cm
OS02878
58 y pT3a pN0 M0 16 cm
KC02831
42 y pT4 pN0 M0 13 cm
KC02543
53 y pT3a pN1 M1 5.7 cm
KC02826
69 y pT2b pN1 M1 29 cm
OS00953
47 y pT3a pN1 M1 11.5 cm
OS03074
60 y pT3b pN0 M1 15.5 cm
KC00236
73 y pT3b pN0 M0 6.6 cm
KC02530
49 y pT3a pNX M0 8.5 cm
Normal
Group 1
Group 2
Group 3

**Figure S1 con't**

**C**

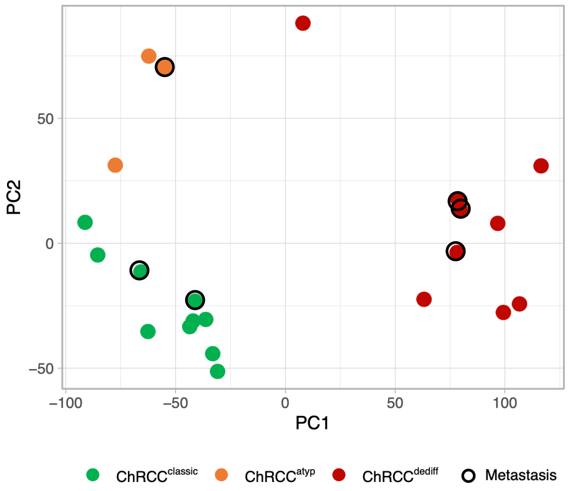


**D**

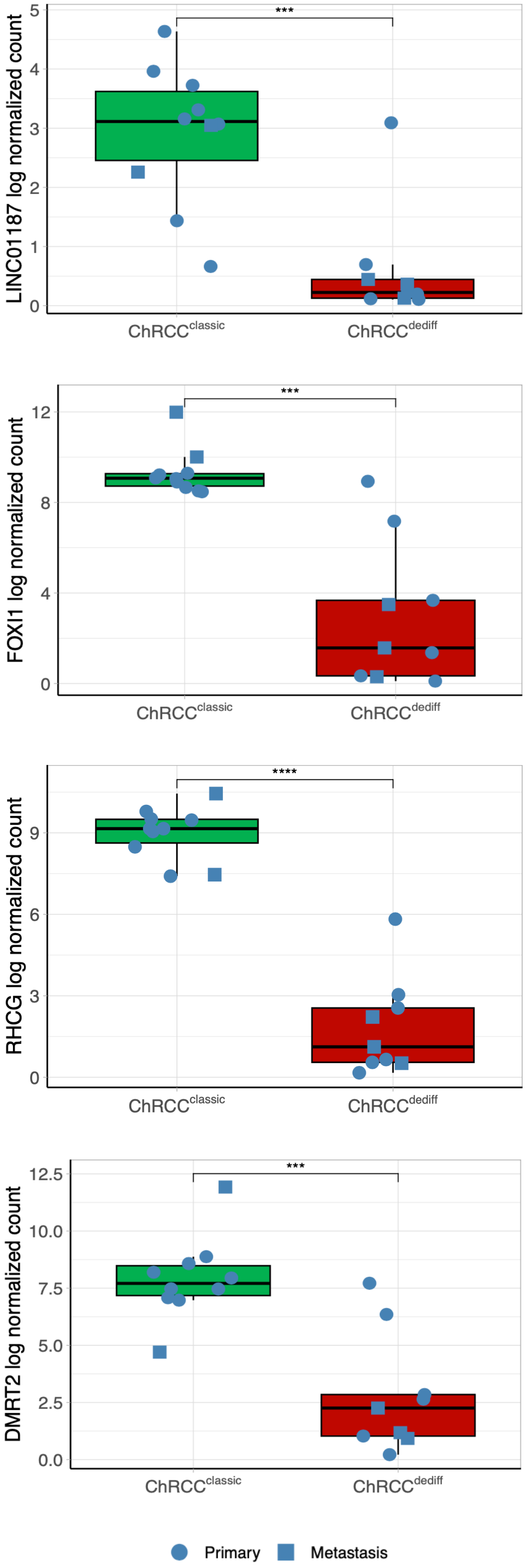


**E**

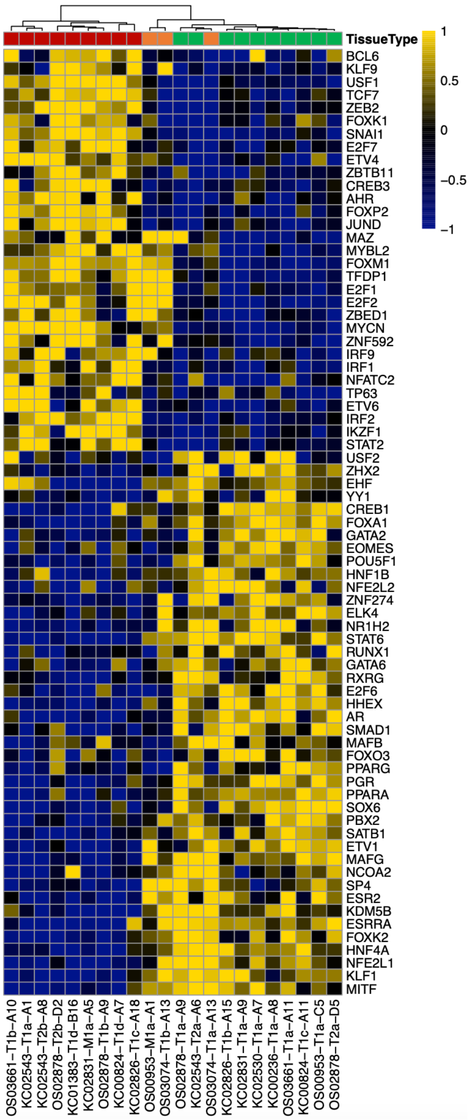

**Figure S2**

**A**

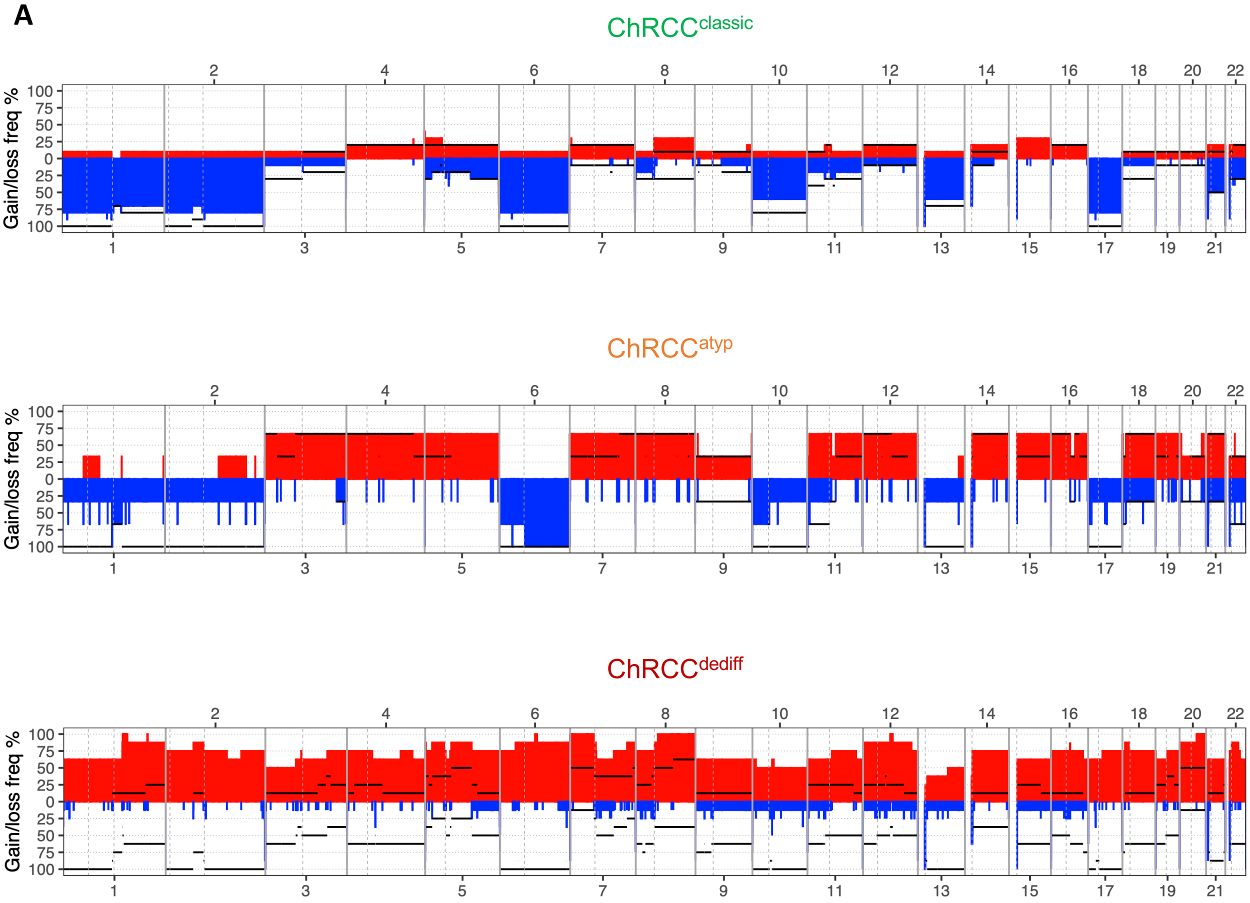
ChRCC^classic
ChRCC^atyp
ChRCC^dediff
Gain/loss freq %
100
75
50
25
0
25
50
75
100
2
4
6
8
10
12
14
16
18
20
22
1
3
5
7
9
11
13
15
17
19
21

**Figure S2 con't**

**B**

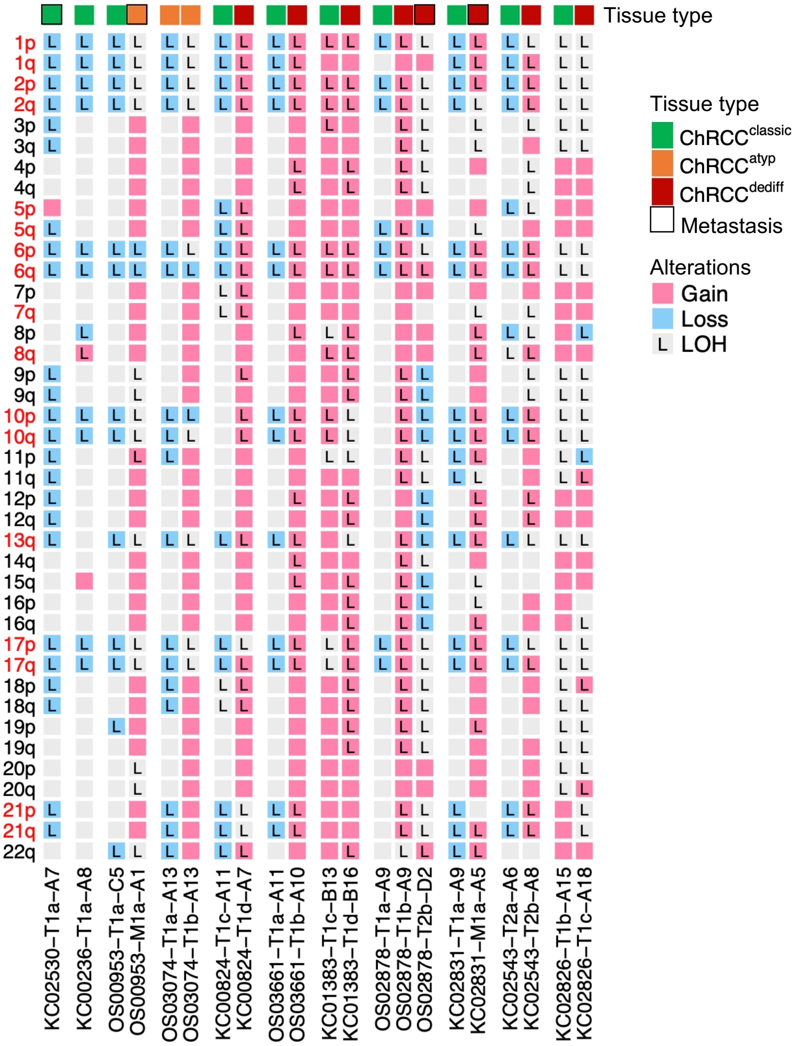

# Figure S2 con't

## C i

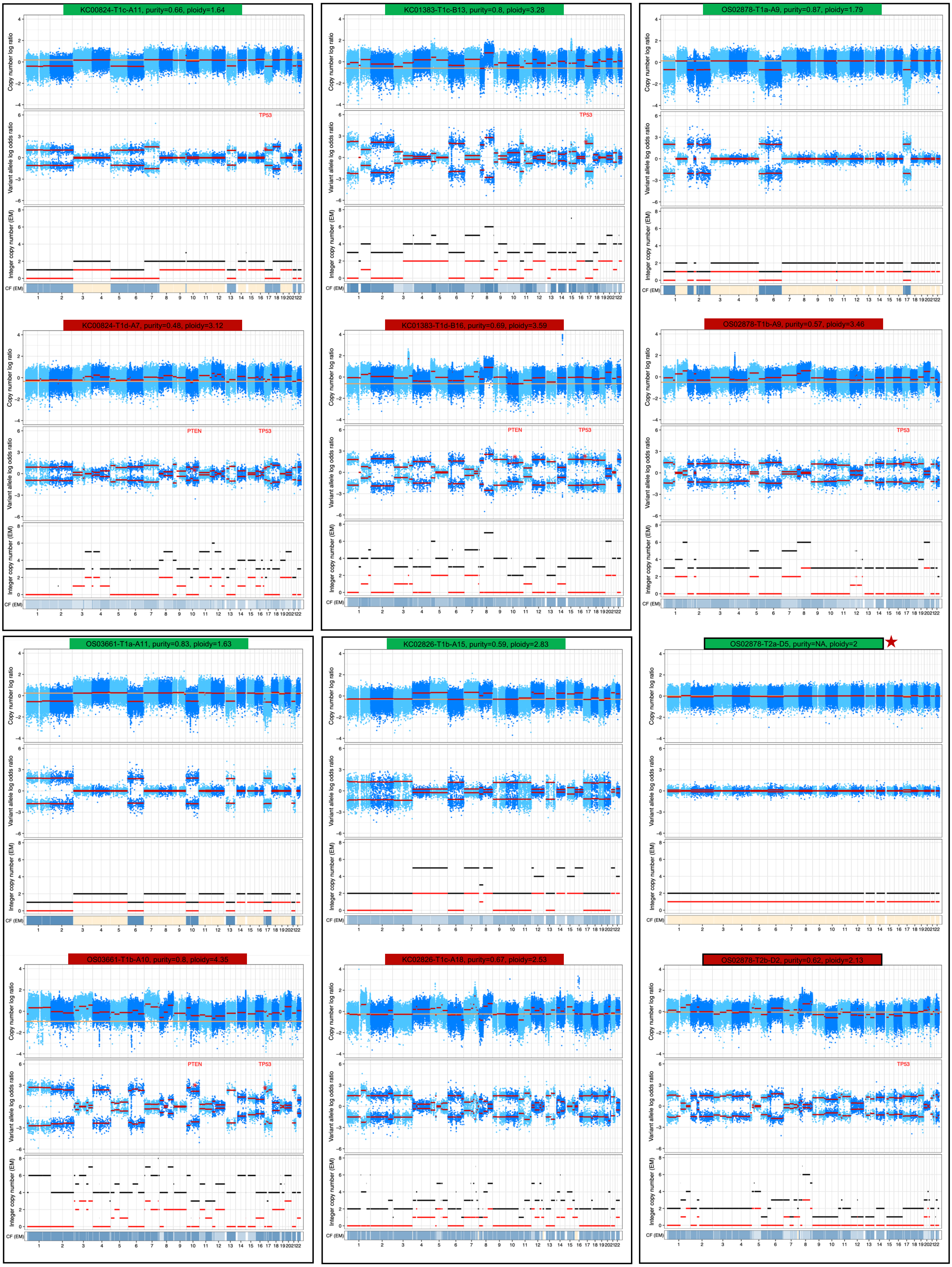

# Figure S2 con't
## C

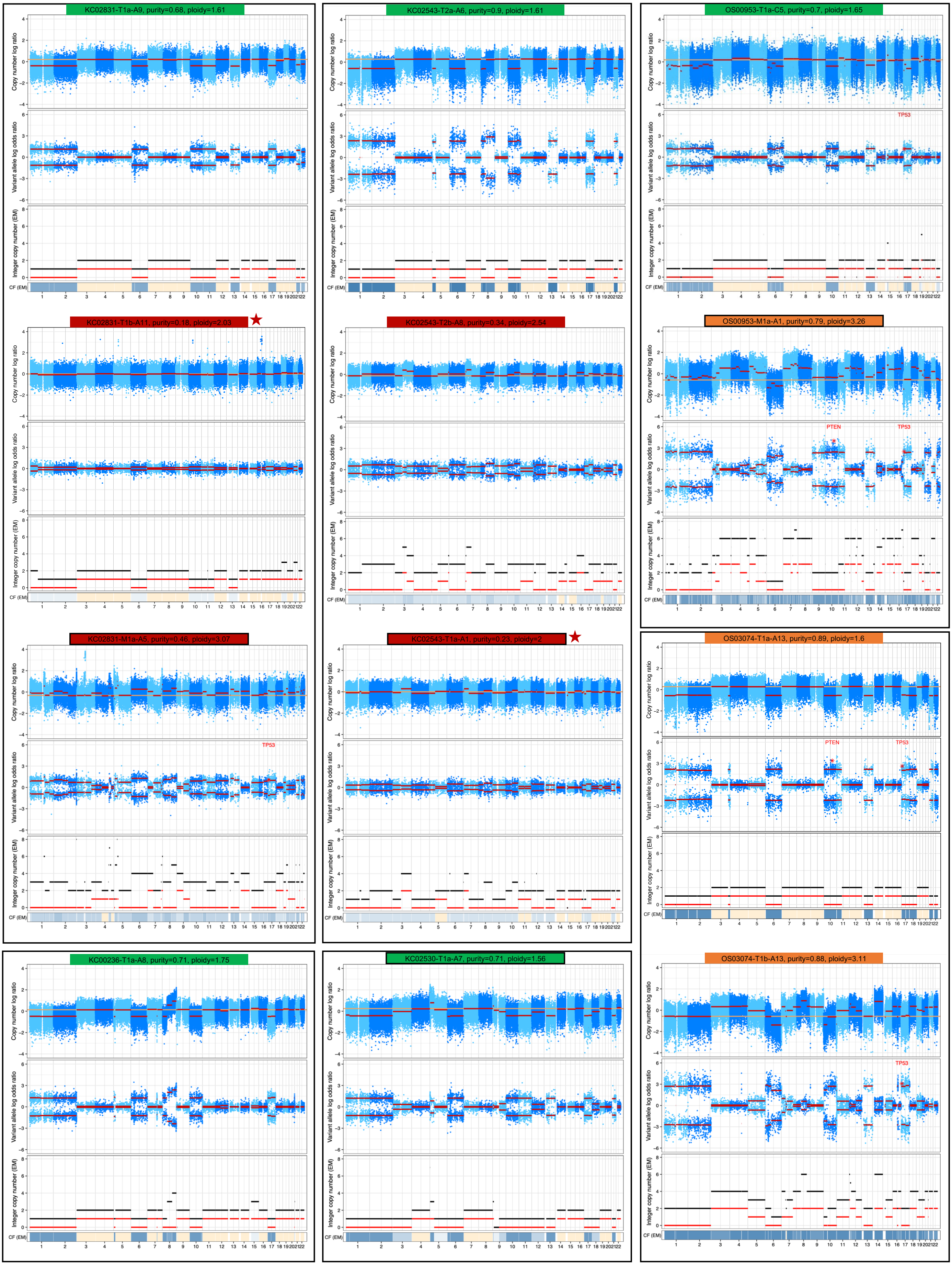

Figure S3

A

KC01383

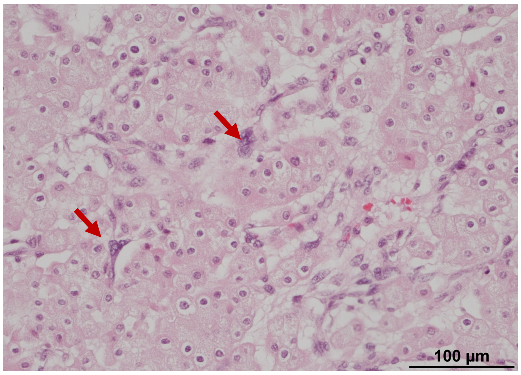


B

| Sample | Histology | Amino Acid Alteration | Variant Classification |
|---|---|---|---|
| OS03661-T1b-A10 | Dedifferentiated | p.X126_splice | Splice Site |
| KC02831-M1a-A5<br>OS00953-M1a-A1<br>OS03074-T1b-A13<br>OS00953-T1a-C5 | Dedifferentiated<br>Atypical<br>Atypical<br>Classic | p.R175H | Missense Mutation |
| KC01383-T1d-B16<br>KC01383-T1c-B13 | Dedifferentiated<br>Classic | p.R213L | Missense Mutation |
| OS02878-T1b-A9<br>OS02878-T2b-D2 | Dedifferentiated<br>Dedifferentiated | p.R249W | Missense Mutation |
| KC00824-T1d-A7<br>KC00824-T1c-A11 | Dedifferentiated<br>Classic | p.V274D | Missense Mutation |
| OS03074-T1a-A13 | Atypical | p.R306* | Nonsense Mutation |

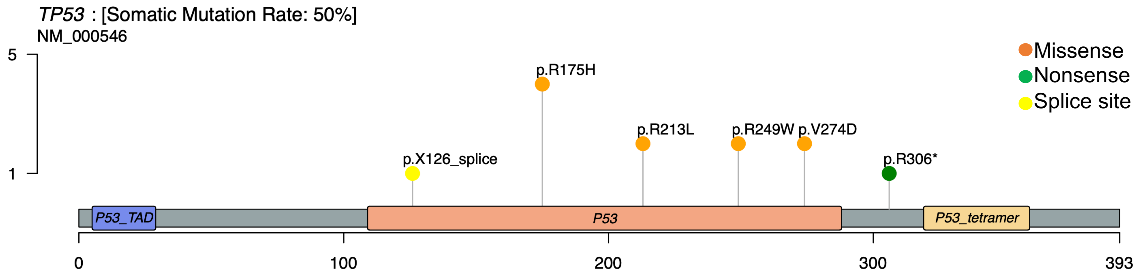


C

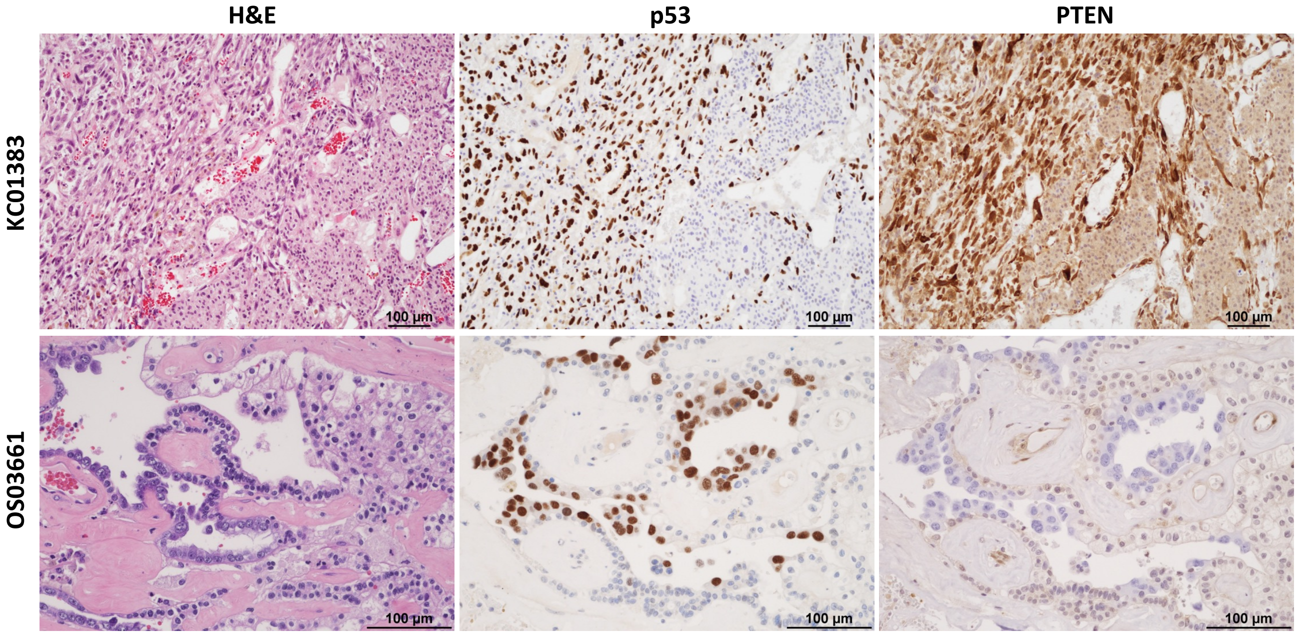

**Figure S3 con't**

**D**

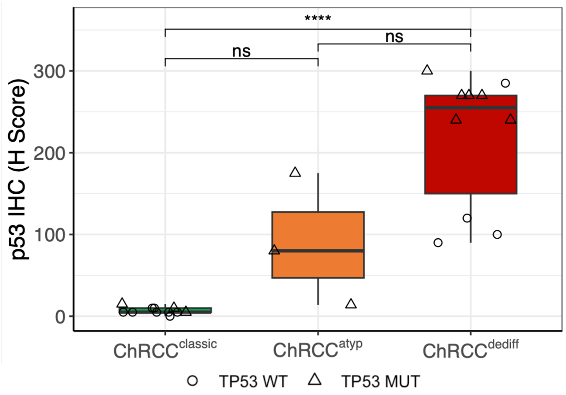


**E**

| Sample | Histology | Amino Acid Alteration | Variant Classification |
|---|---|---|---|
| KC00824-T1d-A7 | Dedifferentiated | p.C124S | Missense Mutation |
| OS03661-T1b-A10 | Dedifferentiated | p.Q171* | Nonsense Mutation |
| KC01383-T1d-B16 | Dedifferentiated | p.E358Q | Missense Mutation |
| OS00953-M1a-A1 | Atypical | p.G165E | Missense Mutation |
| OS03074-T1a-A13 | Atypical | p.X70_splice | Splice Site Deletion |
| OS03074-T1b-A13 | Atypical | p.T319* | Frame Shift Deletion |

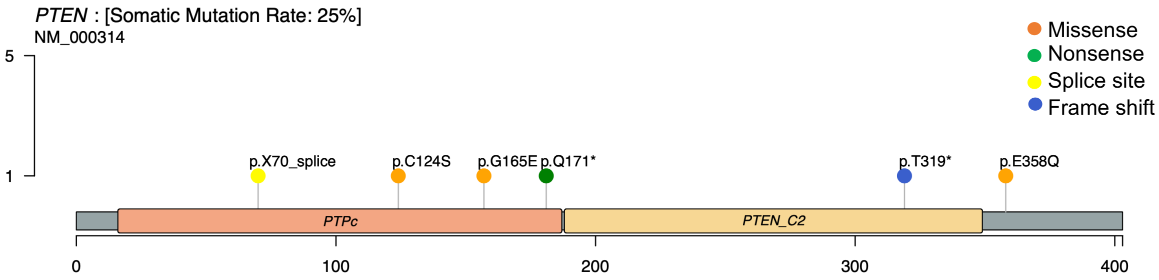

Figure S4

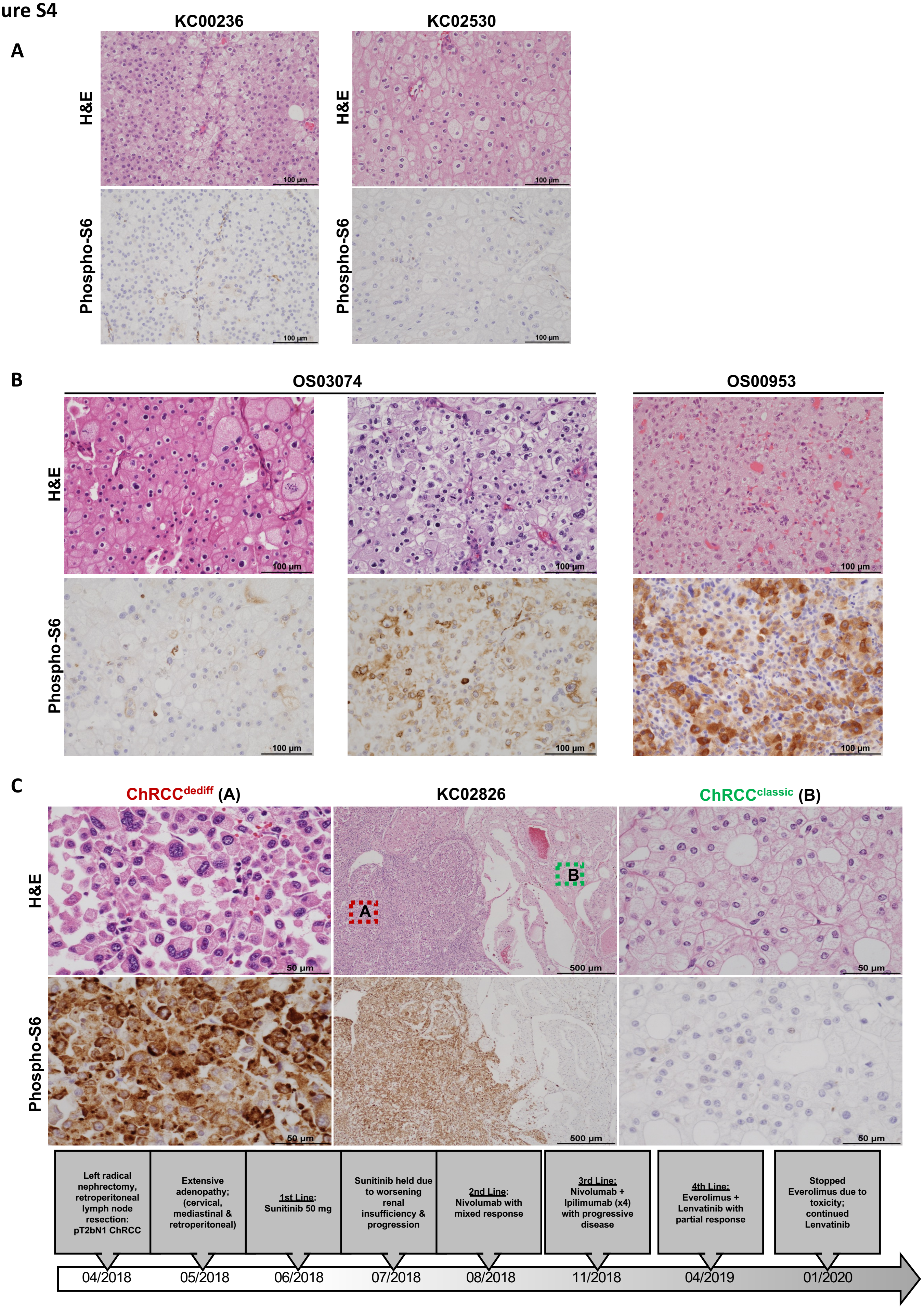
A
KC00236
KC02530
H&E
Phospho-S6
100 µm
B
OS03074
OS00953
H&E
Phospho-S6
100 µm
C
ChRCC^dediff (A)
KC02826
ChRCC^classic (B)
H&E
Phospho-S6
50 µm
500 µm
A
B
Left radical nephrectomy, retroperitoneal lymph node resection: pT2bN1 ChRCC
Extensive adenopathy; (cervical, mediastinal & retroperitoneal)
1st Line: Sunitinib 50 mg
Sunitinib held due to worsening renal insufficiency & progression
2nd Line: Nivolumab with mixed response
3rd Line: Nivolumab + Ipilimumab (x4) with progressive disease
4th Line: Everolimus + Lenvatinib with partial response
Stopped Everolimus due to toxicity; continued Lenvatinib
04/2018
05/2018
06/2018
07/2018
08/2018
11/2018
04/2019
01/2020

# Figure S5

**A**

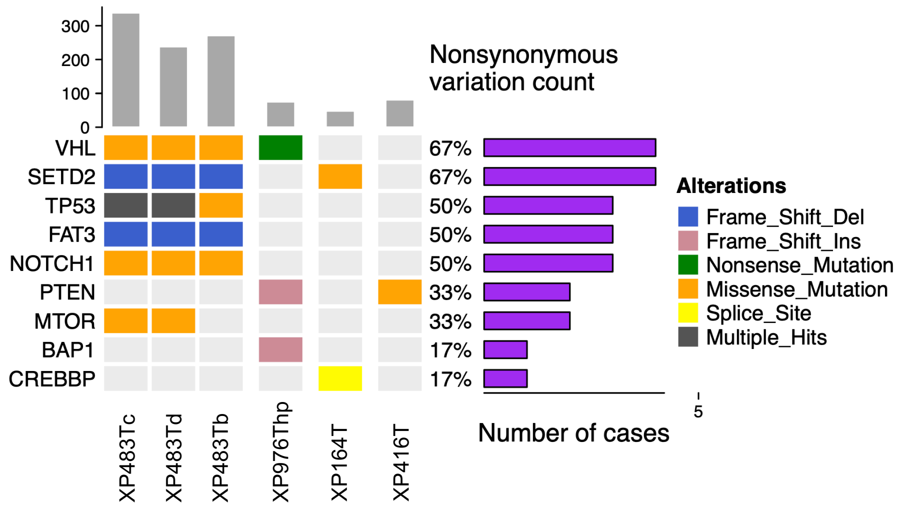


**B**

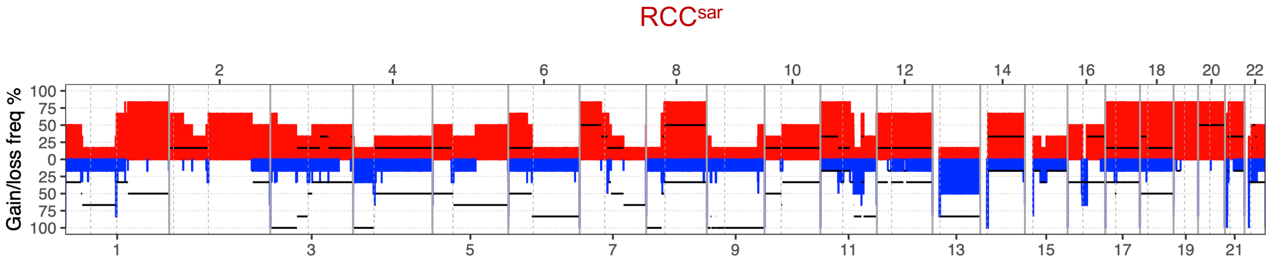


**C**

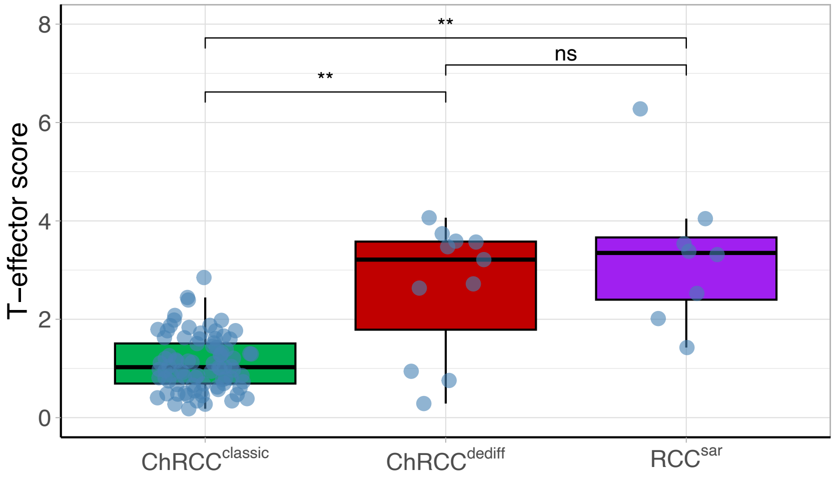

Supplement: Supplemental data [file jciinsight-9-176743-s287.pdf]
